# A DARPin targeting activated Mac-1 is a novel diagnostic tool and potential anti-inflammatory agent in myocarditis, sepsis and myocardial infarction

**DOI:** 10.1007/s00395-021-00849-9

**Published:** 2021-03-15

**Authors:** Patrick M. Siegel, István Bojti, Nicole Bassler, Jessica Holien, Ulrike Flierl, Xiaowei Wang, Philipp Waggershauser, Xavier Tonnar, Christopher Vedecnik, Constanze Lamprecht, Ivana Stankova, Tian Li, Thomas Helbing, Dennis Wolf, Nathaly Anto-Michel, Lucia Sol Mitre, Julia Ehrlich, Lukas Orlean, Ileana Bender, Anne Przewosnik, Maximilian Mauler, Laura Hollederer, Martin Moser, Christoph Bode, Michael W. Parker, Karlheinz Peter, Philipp Diehl

**Affiliations:** 1 Cardiology and Angiology I, Heart Center Freiburg University, Faculty of Medicine, University of Freiburg, Freiburg, Germany; 2 Atherothrombosis and Vascular Biology Laboratory, Baker Heart and Diabetes Institute, Melbourne, Australia; 3 ACRF Rational Drug Discovery Centre, St. Vincent’s Institute of Medical Research, Melbourne, Australia; 4 Baker Department of Cardiometabolic Health, University of Melbourne, Melbourne, Australia; 5 Department of Medicine, Central Clinical School, Monash University, Melbourne, Australia; 6 BIOSS Centre for Biological Signalling Studies/Synthetic Biology of Signalling Processes, University of Freiburg, Freiburg, Germany; 7 Bio21 Molecular Science and Biotechnology Institute, University of Melbourne, Melbourne, Australia; 8 Baker Heart and Diabetes Institute, 75 Commercial Road, Melbourne, VIC 3004 Australia

**Keywords:** DARPin, Inflammation, Myocarditis, Myocardial infarction, ECMO, Sepsis

## Abstract

The monocyte β_2_-integrin Mac-1 is crucial for leukocyte–endothelium interaction, rendering it an attractive therapeutic target for acute and chronic inflammation. Using phage display, a Designed-Ankyrin-Repeat-Protein (DARPin) was selected as a novel binding protein targeting and blocking the α_M_ I-domain, an activation-specific epitope of Mac-1. This DARPin, named F7, specifically binds to activated Mac-1 on mouse and human monocytes as determined by flow cytometry. Homology modelling and docking studies defined distinct interaction sites which were verified by mutagenesis. Intravital microscopy showed reduced leukocyte–endothelium adhesion in mice treated with this DARPin. Using mouse models of sepsis, myocarditis and ischaemia/reperfusion injury, we demonstrate therapeutic anti-inflammatory effects. Finally, the activated Mac-1-specific DARPin is established as a tool to detect monocyte activation in patients receiving extra-corporeal membrane oxygenation, as well as suffering from sepsis and ST-elevation myocardial infarction. The activated Mac-1-specific DARPin F7 binds preferentially to activated monocytes, detects inflammation in critically ill patients, and inhibits monocyte and neutrophil function as an efficient new anti-inflammatory agent.

**Supplementary Information:**

The online version contains supplementary material available at 10.1007/s00395-021-00849-9.

## Introduction

Cardiovascular diseases remain the leading causes of morbidity and mortality worldwide [7]. Activated leukocytes are not only the drivers of vascular inflammation, which is the underlying cause of cardiovascular diseases such as atherosclerosis, but also of many other inflammatory diseases such as sepsis, arthritis, and auto-immune disease [81]. Adhesion molecules, and in particular leukocyte β_2_-integrins, play a major role in the interaction of leukocytes with the endothelium, which is an important first step in leukocyte recruitment to inflamed tissues.

Mac-1 (α_M_ß_2_, CD11b/CD18, complement receptor 3) is an important member of the β_2_-integrin family and is highly expressed on monocytes and neutrophils. It interacts with multiple ligands including fibrinogen, C3bi, ICAM-1, and heparin, and is involved in leukocyte adhesion to and migration through the endothelium, phagocytosis, and the innate and adaptive immune system [59]. Like all β_2_-integrins, Mac-1 is composed of an α-chain (165 kD) and a β-chain (95 kD) [75]. The ectodomains of integrins can adopt a resting and an activated conformation [82]. Cellular activation results in a conformational change of the resting receptor to the activated conformation (via a switchblade mechanism), resulting in exposure of activation-specific ectodomains which mediate high affinity of the activated receptor to several receptor ligands [40]. This activation-specific epitope of the α-chain of Mac-1, the so-called inserted domain (I-domain), thereby mediates several receptor–ligand interactions [20].

Traditional untargeted anti-inflammatory drugs such as steroids or methotrexate have not shown convincing results in cardiovascular diseases and are associated with severe, potentially life-threatening side effects [49]. This imbalance between beneficial effects and side effects may be related to the fact that these anti-inflammatory drugs do not target specific receptor functions but, rather, affect overall leukocyte function. Biologicals such as antibody drugs, in selectively targeting specific cell surface receptors, promise a better ratio of therapeutic effects to nonspecific side effects. A large range of successful antibody drugs targeting surface receptors have been developed as anti-inflammatory and cancer drugs. These drugs bind to specific regions/epitopes in the receptor and can thereby block specific ligand-binding and specific receptor functions [79]. Furthermore, integrin receptors such as Mac-1 are of particular interest for therapeutic targeting as they offer additional specificity by virtue of having an active, ligand-binding conformation. A good example is GPIIb/IIIa where activation-specific antibodies have been shown to avoid classic side effects such as paradoxical platelet activation and prolongation of bleeding time [2]. A similar approach was undertaken for the integrin receptor Mac-1, producing a single-chain antibody (MAN-1) that was specific for the active conformation of Mac-1; however, this antibody did not provide cross-reactivity to the murine Mac-1 and thus could not be tested in a mouse preclinical model and as a consequence was not further developed as a drug candidate [25]. This paper describes a novel approach aiming to develop a biological, highly sought-after anti-inflammatory drug candidate that would allow specific inhibition of the activated conformation of Mac-1.

Designed Ankyrin Repeat Proteins (DARPins) are a novel class of binding molecules derived from naturally occurring ankyrin repeat proteins [69]. They can be selected against multiple types of targets using phage or ribosome display [60]. DARPins offer several advantages over antibody drugs and small binding molecules, such as very high stability over a wide range of temperatures and pH values [78], the possibility of using bacterial expression systems with particularly high yields, ease of purification, and potential large-scale production [9]. Their small size (≈14–21 kD) may also be of advantage in diagnostic or therapeutic applications where increased tissue penetration is required, e.g. tumour therapy, inflammation, and imaging [10]. Due to these advantages, as well as high affinity and specificity binding to their target epitopes, DARPins are very attractive as diagnostic tools and therapeutic agents, and in fact the first DARPins have been successfully established as drugs in clinical applications such as neovascular age-related macular degeneration [50].

We aimed to select a DARPin targeting the ligand-binding site of the β_2_-integrin Mac-1 and thereby achieve activation-specific blocking of Mac-1, which is a key player in cardiovascular inflammation [80]. A DARPin targeted to the I-domain should block ligand interaction without affecting the function of the resting integrin. This approach would deliver very selective inhibition of the highly pro-inflammatory subpopulation of activated leukocytes, without inhibiting the non-activated majority of leukocytes. As such, we describe an innovative drug candidate that is potentially clinically applicable in a broad range of diseases with inflammatory components.

## Methods

### ***Mouse α***_***M***_*** I-domain and human α***_***M***_*** I-domain***

To select DARPins against the mouse α_M_ I-domain, a DARPin-expressing phage library consisting of 10^10^ phages was used [67]. To assess for cross-reactivity, binding of selected anti-α_M_ I domain (anti-mId) DARPins to the human α_M_ I-domain was investigated in Enzyme-Linked Immunosorbent Assays (ELISA). According to the PubMed data bank, the following sequence was used for the mouse α_M_ I-domain: MVLRPPQQFPEALRECPQQESDIVFLIDGSGSINNIDFQKMKEFVSTVMEQFKKSKTLFSLMQYSDEFRIHFTFNDFKRNPSPRSHVSPIKQNGRTKTASGIRKVVRELFHKTNGARENAAKILVVITDGEKFGDPLDYKDVIPEADRAGVIRYVIGVGNAFNKPQSRRELDTIASKPAGEHVFQVDNFEALNTIQNQLQEKIFAIEGTQTGSTSSFEHEMSQEGFSASRGGPEQKLISEEDLNSAVDLPETGGEAAALEHHHHHH.

For the human α_M_ I-domain, the following sequence was used: RQQPQKFPEALRGCPQEDSDIAFLIDGSGSIIPHDFRRMKEFVSTVMEQLKKSKTLFSLMQYSEEFRIHFTFKEFQNNPNPRSLVKPITQLLGRTHTATGIRKVVRELFNITNGARKNAFKILVVITDGEKFGDPLGYEDVIPEADREGVIRYVIGVGDAFRSEKSRQELNTIASKPPRDHVFQVNNFEALKTIQNQLREKIFAIEGTQTGSSSSFEHEMSQEGFSAAALEHHHHHH.

The human α_M_ I-domain was cloned into the bacterial expression vector pET-20b(+), while the mouse α_M_ I-domain was cloned into the bacterial expression vector pHOG21. Both were expressed in *E. coli BL21 DE3* and proteins were purified as described below. To assess conformation-specific binding of DARPins, a mouse α_M_ I-domain mutant (F302L), as described by Hu et al. [36], which represents the activated α_M_ I-domain conformation (a-mId), was cloned and expressed like the wild-type mouse α_M_ I-domain described above.

The functionality of the recombinant human and mouse α_M_ I-domains was assessed by their binding to human or mouse ICAM-1, respectively, which are physiological ligands of Mac-1. 96-well plates (Maxisorb, Thermo Scientific, USA) were coated with 0.1 μM human or mouse ICAM-1 (R&D Systems, USA) or BSA (Sigma-Aldrich, USA, 30 µM), then recombinant human or mouse α_M_ I-domain binding was quantified with anti-His-HRP staining on an ELISA plate reader at λ 450 nm. The ELISA was designed by the researchers.

### Phage panning using DARPin-expressing phage library

The phage library used in this project contains a very high diversity of approximately 10^10^ and was kindly provided by Molecular Partners (Zurich, Switzerland). This phage diversity allows selection of high-affinity DARPins against most biological antigens. The affinity of the DARPin phagemids is in the nanomolar range [67]. DARPin phagemid particles contain the phagemid vector pMPAG-3, in which the DARPin encoding insert is located in the Multiple Cloning Site (MCS) between the *Bam*HI and *Hind*III restriction sites. pMPAG-3 mediates chloramphenicol (CML) resistance, allowing selective growth of only phagemid-infected *E. coli* cells in CML-containing media/agar plates.

**Fig. 1:**
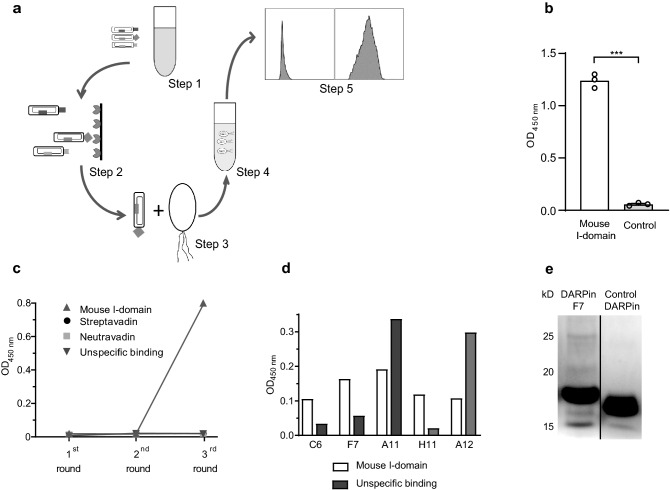
Selection, expression, and purification of DARPin F7**.** **a** DARPin-presenting phages were incubated with the mouse α_M_ I-domain (step 1). Phages bound to the mouse α_M_ I-domain were harvested (step 2), *E. coli* were infected (step 3) and phages were amplified (step 4). After several panning rounds, DARPins of selected phages were produced and tested for their specific binding behaviour by flow cytometry (step 5). Underlying images of **a** were adapted from http://smart.servier.com/ under a creative commons licence. **b** The binding capability of recombinant mouse α_M_ I-domain was assessed with immobilized mouse ICAM-1 compared to a negative control (BSA) in an ELISA (*n *= 3, *p < 0.001). An increase in OD_450nm_ indicates increased binding. The error bars indicate SEM. *p*-value was calculated using an unpaired two-tailed Student’s *t*-test. **c** Polyclonal phages bound to the α_M_ I-domain, demonstrating phage enrichment after 3 panning rounds. **d** After the third phage panning round, several DARPins were screened for their affinity to the mouse α_M_ I-domain in an ELISA. Screening was performed in a 96 well format with one DARPin clone produced in each well. Binding of DARPins to the mouse α_M_ I-domain was then assessed by ELISA. Wells were coated with mouse α_M_ I-domain or PBS. Several representative clones are presented. DARPin clone F7 was chosen due to its stronger binding characteristics to the mouse α_M_ I-domain. **e** Coomassie Brilliant Blue stained 12% polyacrylamide SDS gel showing DARPin F7 and the control DARPin after expression and purification. Expression and purification were performed as described in the "Methods" section. In brief, DARPins were produced in BL21-DE3 bacterial cells. Following bacterial lysis, DARPin purification was carried out using Ni^+^ coated agarose beads, which bound to the DARPins’ His-tag. After several wash steps purified DARPins were obtained. Gels were loaded with 20 µg of the final elution of DARPins. The molecular weight of F7 and the control DARPin is around 17 kD. The lanes were run on the same gel but are non-contiguous. Before loading proteins onto the SDS gel, DARPins were denatured by addition of a denaturing sample buffer containing 10% SDS and heating to 95 °C for 5 min. A full unedited gel is provided as Online Resource 18

DARPin phages were selected on a soluble and immobilized α_M_ I-domain using phage display standard protocols (Fig. 1a) [15]. Briefly, streptavidin- or neutravidin-coated magnetic beads were blocked with an albumin- and TWEEN^®^-containing buffer. After removal of DARPins that bound unspecifically to beads, phages were incubated (0.1 μM, 1 h, wheel, RT) with biotinylated α_M_ I-domain, then phage I-domain complexes were harvested using streptavidin- or neutravidin-coated beads. After several washing steps, phages bound to the I-domain were eluted with a buffer containing glycine (200 mM, pH 2) and samples were neutralized with tris/base (2 M) before being used for infection with log-phase bacteria. Infected *E. coli* were grown for 30 min without shaking at 37 °C followed by 30 min at 220 rpm (37 °C) before cells were plated out on CML-containing agar plates (overnight, 37 °C). Colonies were harvested and grown in CML-containing media until OD_600_ reached 0.5, then the culture transferred into LB medium containing CML, isopropyl-β-d-thiogalactopyranosid (IPTG; Merck, Germany), and helper phages (overnight, 37 °C). Phagemid particles from the overnight culture were purified by spinning (16,000*g*, 4 °C, 20 min) and incubating the supernatant with polyethylenglycol (PEG; Sigma Aldrich, USA) on ice for > 1 h. PEG-purified phages were concentrated and used for the next phage-panning round.

### Polyclonal phage ELISA

To assess the affinity enrichment of the selected phages, a polyclonal phage ELISA was performed after three panning rounds. The biotinylated α_M_ I-domain was placed into neutravidin-coated ELISA wells and phages from the corresponding panning rounds were added. Binding of phages to α_M_ I-domain-coated wells was assessed with an anti-M13 HRP-coupled antibody (100 µl at 1:40,000 dilution, 4 °C, 1 h; GE Lifescience, USA).

### Cloning of DARPins from phage vector into expression vector

DARPins from the third panning round in the phage vector (pMPAG-3) were digested with *BamH*I and *Hind*III (1 h, 37 °C), DARPin-encoding inserts were gel-purified and cloned into the expression vector pMPAG-6 (Molecular Partners, Switzerland). DARPins in pMPAG-6 were transformed into TG-1 cells and the binding behaviour of each transformed clone assessed in a monoclonal DARPin ELISA coated with the α_M_ I-domain.

### ***Production and purification of DARPins and α***_***M***_*** I-domain***

Expression vectors contained a T5 promotor allowing high-level expression of the DARPins or human and mouse α_M_ I-domains in *E. coli* cells using IPTG. Furthermore, the vectors encode for ampicillin resistance, allowing selective growth in ampicillin-containing media. DARPins applied in mouse models contained an albumin binding domain, which by its binding to circulating albumin is associated with a half-life in vivo of approximately 1–2 days [68]. We used Luria Broth (LB) medium. After overnight culturing (10 ml in a 50 ml conical tube, 12 h, 37 °C, 220 rpm), clones were grown in LB medium (0.5–1 l; Sigma Aldrich, USA) containing ampicillin (*F*_c_ = 100 µg/ml) until OD_600_ of 0.4–0.7 was reached. After induction with IPTG (*F*c = 500 µM), cultures were grown for 5 h (DARPins) or overnight (I-domain). Cells were then harvested (20 min, 4000 g, 8 °C) and stored at −80 °C until purification. AVB 101 cells (Avidity AVItag™, Colorado, USA) were used for expression of biotinylated DARPins enabling in vivo biotinylation of proteins according to the manufacturer’s instructions.

Frozen bacterial pellets were resuspended in Bugbuster Mastermix (Merck, Germany) as recommended by the manufacturer. Recombinant proteins were separated from cellular debris by centrifugation (15 min, 16,000*g*, 4 °C), captured by Ni^+^ beads (Qiagen, Germany), washed five times with wash buffers (10 and 20 mM imidazole, Merck, Germany) and eluted with the elution buffer (250 mM imidazole). Eluted recombinant proteins were dialyzed against 2 l of PBS for at least 24 h and used for further experiments.

### SDS- and Western blotting of recombinant proteins

Size and purity of recombinant proteins were assessed in 12% sodium dodecylsulfate polyacrylamide electrophoresis (SDS-PAGE) with Coomassie staining and Western blotting using standard protocols. For Western blots of DARPins, gels were blotted on a polyvinylidene fluoride (PVDF) membrane (Immobilon, Merck Millipore, Germany) before the membrane was blocked with 3% BSA (30 min, 70 rpm, RT). Blotted DARPins and the α_M_ I-domain were detected using an anti-His-Peroxidase antibody (1:8000; Roche Diagnostics, Switzerland) with SuperSignal ECL reagent (Thermo Scientific, USA).

### Flow-cytometric binding assays

DARPin binding to the mouse monocytic cell line RAW 264.7 (ATCC, USA) was determined as follows: 5 × 10^5^ cells were resuspended in 100 µl PBS + Ca^2+^ (0.88 mM) and Mg^2+^ (0.49 mM) and stimulated with phorbol 12-myristate 13-acetate (PMA, *F*_c_ = 200 ng/ml, 15 min, 37 °C) or PBS + Ca^2+^ and Mg^2+^ as a negative control. RAW 264.7 cells were stained with a FITC anti-CD11b antibody (Clone M1/70, BD, USA), an isotype control antibody or DARPins (*F*_c_ = 2.5 µg/ml). Gating was performed according to FSC/SSC plots (Online Resource 1).

DARPin binding to monocytes was also assessed in whole mouse or human blood. 100 µl freshly obtained citrated blood was stimulated with PMA, (*F*_c_ = 200 ng/ml, 15 min, 37 °C) or PBS + Ca^2+^ and Mg^2+^ as negative control, followed by red blood cell lysis (mouse blood: 5 min lysis, human blood: 20 min lysis, on ice; BD FACS lysing solution, USA). Samples were centrifuged (300*g*, 5 min), washed and resuspended in 100 µl PBS + Ca^2+^ (0.88 mM) and Mg^2+^ (0.49 mM) + 0.1% BSA.

After cells were stained with DARPins for 15 min, mouse monocytes were stained with a Pacific Blue anti-Ly-6C antibody (10 µl, 1:50 dilution; BD Biosciences, USA), DARPins were detected with an Alexa Fluor 488 anti-His-tag antibody (1 µl undiluted; Qiagen, Germany) and cells were fixed (BD CellFIX™, USA). For mouse monocytes, DARPin binding to Ly-6C^++^ monocytes was investigated. For human monocytes Pacific Blue anti-CD16 and APC anti-CD14 antibodies (both: 5 µl, 1:10 dilution; Biolegend, USA) were used for staining and gating and binding to CD14^+^ cells was assessed. FACS was performed within 2 h of sample preparation to prevent degradation of monocyte surface markers.

To investigate specific binding of DARPin F7 to the activated Mac-1 receptor, competitive binding assays of F7 versus conventional anti-Mac-1 antibodies were performed. The protocol was performed as described above with the following alterations: PMA-stimulated leukocytes from freshly obtained human blood were pre-incubated (15 min, RT) with anti-Mac-1 antibodies M1/70 (*F*_c_ = 50 µg/ml) (Biolegend, USA) or Mab107 (*F*_c_ = 10, 50, 100 µg/ml) (Absolute Antibody, USA) at different concentrations. Then DARPins (*F*_c_ = 2.5 µg/ml, 15 min, ice) were added and the protocol was performed as described above.

Activation-specific binding of F7 to activated human CD14^+^ monocytes was also assessed compared to other anti-CD11b antibodies for visual analysis of the “population shift” in the FITC-histogram occurring after PMA-stimulation. Whole blood was prepared and monocytes were stained as described above. CD11b expression and conformational change was detected either with DARPin F7, the scFv MAN-1 (kindly provided by Prof. Karlheinz Peter) which were detected by an Alexa Fluor 488 anti-His-tag antibody (Qiagen, USA) or the following anti-Mac-1 antibodies (*F*_c_ = 10 µg/ml): Mab107 (Absolute Antibody, USA), 2LPM19c (Santa-Cruz Biotechnology, USA), M1/70, Mab24, CBRM1/5 (all from Biolegend, USA). Antibody binding was detected by appropriate FITC-conjugated secondary antibodies (1 µl, 15 min, RT).

In addition to competitive binding assays, binding competition between recombinant ICAM-1 (rICAM; Biolegend, USA), which is a physiological ligand of Mac-1, and F7 was conducted. Monocytes were isolated from healthy C57BL/6N mice using a monocyte isolation kit (STEMCELL, France) and stained with F7, PBS or the control DARPin (15 min, RT) followed by incubation with rICAM (15 min, RT). ICAM-1-binding was detected by a FITC anti-ICAM-1 antibody (Biolegend, USA).

When a histogram showed a single-peak monocyte population, the mean fluorescence intensity (MFI) was used as a read-out for DARPin binding (detected by anti-His-tag antibody). When more than one peak in the gated final monocyte population was visible DARPin binding was quantified in percentage. To define the positive population, a gate was set in the FITC histogram including the upper 1–3% of the unstimulated monocyte population stained with the secondary anti-His antibody. The monocytes stained with DARPins and the secondary antibody shifting into this gate were then recorded in percentages.

For assessment of human neutrophils, approximately 5 × 10^6^ neutrophils were isolated by density gradient centrifugation from citrated human whole blood using Polymorphprep^©^ (Alere Technologies, Norway). Neutrophils were identified by Pacific-Blue anti-CD66b antibody staining (5 µl; 15 min before acquisition, RT; Biolegend, USA) in the Pacific Blue/SSC gate and verified by back-gating to the FSC/SSC plot. Mean FITC fluorescence intensity was used to monitor DARPin binding. DARPins (*F*_c_ = 2,5 µg/ml) and the secondary antibody (1 µl, Alexa Fluor 488 anti-His-Tag antibody, Qiagen, Germany) were added after 2 min of acquisition. To stimulate neutrophils, PMA was added after 20 min. Acquisition was stopped after 40 min. The mean percentage change from baseline of the geometric mean fluorescence is presented. Baseline was determined by calculating the average FITC MFI during the period after addition of DARPins (*t* = 2 min) until stimulation with PMA (*t* = 20 min).

The gating strategies described above and the gating strategy for the identification of leukocyte subsets by flow cytometry in the murine experimental autoimmune myocarditis and cecal ligation and puncture model are described in the Online Resource 1.

Acquisition of all FACS data was performed using a BD FACS Canto II Flow Cytometer and FACS DIVA software. Data was analyzed with FACS DIVA V6.1 (Becton Dickinson, USA) and FlowJo^©^ V10 software.

### Isolation of mouse cardiomyocytes

Isolation of murine cardiomyocytes was performed as described elsewhere [57]. Briefly, after removal, the murine heart was connected to a Langendorff perfusion system and perfused with collagenase containing buffers up to 50 min. After digestion, the heart was transferred into petri dishes, the atria were removed, and undigested parts of the heart muscle were eliminated using a filter. Isolated cardiomyocytes were then cultured in laminin-coated cell-culture dishes until used for experiments.

### Quantitative real-time PCR

Expression of Mac-1 in isolated monocytes and expression of ICAM-1 in isolated cardiomyocytes were assessed by quantitative real-time PCR (qRT-PCR). RNA from monocytes was isolated using the Aurum™ Total RNA Mini Kit (Bio-Rad, USA) within 30 min of isolation of primary mouse monocytes and after 24 h starvation of these monocytes (medium not supplemented with FCS). Cardiomyocyte-RNA was isolated within 30 min of cardiomyocyte isolation from whole murine hearts and after 4 h stimulation of these cardiomyocytes with mouse TNFα (10 ng/ml). Primers were obtained from Bio-Rad (Prime PCR™). QRT-PCR was performed according to standard protocols using SYBR Green (Bio-Rad, USA).

### Single-cell force spectroscopy of monocyte–cardiomyocyte interaction

To investigate whether F7 blocks the interaction of Mac-1 on monocytes with ICAM-1 expressed on cardiomyocytes, atomic force microscopy (AFM) based single-cell force spectroscopy (SCFS) was used, which is an established method to quantify cell–cell interaction forces [8, 34]. Monocytes were isolated from healthy C57BL/6N mice using a monocyte isolation kit (STEMCELL, France). Prior to SCFS mouse monocytes were stimulated with PMA (1 ng/ml, 15 min, 37 °C) or PBS (negative control) and primary mouse monocytes were stimulated with mouse TNFα (10 ng/ml, 1 h, 37 °C), incubated with either F7 or control DARPin (5 µg/ml, 15 min), attached to the cantilever of the AFM, and pressed onto cultured primary mouse cardiomyocytes. The detachment force (*F*_detach_ (pN)) of monocytes attached to cardiomyocytes was recorded and used for statistical analysis.

### Mutagenesis analysis

Homology models were created of F7 and the mouse α_M_ I-domain using SWISS-MODEL (http://swissmodel.expasy.org). The template for the mouse α_M_ I-domain was the I-domain of human Mac-1 [5] and the template for F7 was the crystal structure of a DARPin in complex with AcrB [55]. The quality of the models was evaluated using the SWISS-MODEL Structure Assessment Tool (https://swissmodel.expasy.org/assess). The standard parameters of ZDOCK (http://zdock.umassmed.edu) were used to initially dock both models together. The top 10 DARPin F7: mouse α_M_ I-domain complexes were analysed, with the lowest-energy complex of the largest docking cluster selected for more comprehensive docking using RosettaDock (Lyskov S., Gray J.J. “The RosettaDock server for local protein–protein docking” Nucleic Acids Research 36 (Web Server Issue), W233–W238 (2008)). The ten lowest-energy solutions were downloaded and visually analysed using the PyMOL Molecular Graphics System (Version 1.6.0.0, Schrodinger, USA). It was found that these ten solutions could be grouped into two clusters with very similar orientations. The lowest energy solutions of each cluster were visually analysed and led to two distinct models: “first model” and “second model”. Mutations in the mouse α_M_ I-domain were conducted by “site-directed mutagenesis”. As the designated plasmids are usually very long, a proofreading polymerase (Phusion^©^, New England Biolabs, USA) was used. Due to repetitive sequences, mutations in DARPin F7 were created by cloning a DNA sequence containing the desired mutations (GeneArt™, Germany) into the expression vector. Successful mutagenesis was verified by sequencing. The location of the mutations within the DNA and protein sequences of the mouse α_M_ I-domain and DARPin F7 are presented in the Online Resources 2 and 3.

Binding of mutant F7 to mouse and human α_M_ I-domains was assessed using ELISA. Wells were coated with 100 µl target antigen (0.1 µM, 4 h, 37 °C) per well (mouse α_M_ I-domain wild type or mutants, human α_M_ I-domain wild type), blocked with BSA (1%, 1.5 h, 37 °C), washed 6× with TBS-T, and incubated with 100 µl DARPins (*F*_c_ = 1 µg/ml if not otherwise specified, 1 h, 37 °C). After washing, Streptavidin-HRP (1:2000, 1 h, 37 °C; BD Biosciences, USA) followed by TMB substrate (50 µl/well) was added for visualisation.

### Endotoxin removal

Prior to use, endotoxins were removed based on phase separation with Triton X-114 (Sigma-Aldrich, Germany) using standard protocols. Content of endotoxins was assessed using the LAL chromogenic endotoxin quantification kit (ThermoScientic, USA).

### Intravital microscopy

To investigate the effect of DARPins on monocyte–endothelial interaction in vivo, a murine cremaster model of intravital microscopy was performed [4, 27]. Systemic inflammation was induced with 200 ng mouse TNFα i.p. (ThermoFisher Scientific, USA) in C57BL/6J mice (25–30*g*) 3 h before surgery. For intravital microscopy, rhodamine dye was given intravenously and thereby circulating cells including leukocytes were made fluorescent and could be visually analyzed under the fluorescence microscope. Mice were anesthetized with ketamine (100 mg/kg) and xylazine (5 mg/kg) before 50 µl rhodamine 6G (0.1% solution with 0.9% NaCl) was applied and the cremaster model was performed. The skin was opened using fine scissors and connective tissue and fat was removed with the fine forceps freeing the cremaster muscle. The cremaster muscle was split using the vascular scissors, spread out and fixed using thread and tape. Care was taken to irrigate the operation site with sterile saline solution during the whole procedure. 100 µg DARPin or an equivalent volume of PBS were injected into the femoral vein, and microscopy and recording were performed immediately (Zeiss Axio Lab. A1 Fluorescence microscope, Zeiss, Germany). Recordings were visually analyzed using the Axio Vision program (Zeiss, Germany). The number of rolling leukocytes per 100 µm vessel wall and the number of adherent leukocytes per 100 µm vessel wall were quantified. Leukocytes not moving for more than 30 s were considered adherent. Rolling velocity was calculated by dividing the distance (100 µm) by the time (in seconds) for different leukocytes to travel 100 µm through the vessel (in µm/s).

### Murine model of cecal ligation and puncture

The anti-inflammatory effect of F7 was further investigated in a murine model of polymicrobial sepsis (cecal ligation and puncture (CLP) model) as described elsewhere [63, 79]. A monitoring time frame of 20 h was approved by the animal ethics review board. Permission for survival analysis was not received. C57BL/6 male wild-type mice (25–30*g*) were treated with saturating dosages of DARPin intravenously by tail-vein injection 1 h before surgery (200 µg F7, 200 µg control DARPin). Following anaesthesia with ketamine/xylazine, the peritoneal cavity was carefully opened. The cecum was located, isolated and externalized using the blunt anatomical forceps. It was ligated about 2 mm distal of the ileo-cecal valve using a non-absorbable 6-0 suture. Care was taken not to ligate the ileocecal valve so as to maintain intestinal continuity. Cecal contents were pushed towards the distal cecum and perforation was performed carefully using a 23-G needle. A small drop of faeces was squeezed through the puncture and the cecum was relocated into the peritoneal cavity avoiding the spread of faeces from the cecum onto the abdominal wall wound margins. The peritoneum, fasciae and abdominal musculature were closed by a simple running suture. The skin was closed using metallic clips. Animals were resuscitated by injecting prewarmed isotonic saline solution (5 ml per 100*g* body weight subcutaneously). Subcutaneous buprenorphine was injected as an analgesic. Mice were placed back into their cage and monitored every 2 h. Mice were euthanized 20 h after surgery and the final harvest performed, which included cardiac puncture for blood-taking and peritoneal lavage, followed by a FACS analysis of different leukocyte subsets. Identification of leukocyte subsets by flow cytometry is described in Online Resource 1.

### Murine myocarditis model

To determine the anti-inflammatory potential of DARPin F7 in myocarditis, we used a well-established mouse model of experimental autoimmune myocarditis (EAM) as previously described [46]. 8-week-old female BALB/c mice (Charles River Laboratories, USA) received freshly mixed 0.1 ml porcine myosin (1 mg/ml) (Sigma-Aldrich, St. Louis, USA) dissolved in 0.1 ml Complete Freund’s Adjuvant (Sigma-Aldrich, St. Louis, USA) subcutaneously on days 0 and 7. Mice were treated with F7, control DARPin, or PBS (Gibco, USA) i.p. (intraperitoneal route) on days 0, 7, 8, 9 in a saturating dosage (200 µg for DARPins, approximately 200 µl of a 1 mg/ml solution). Echocardiography was performed on days 0, 14 and 50. Blood samples were also collected on days 0, 14 and 50. The mice were euthanized on day 50 and histology was performed.

Echocardiography was performed under isoflurane anaesthesia (4 Vol%, AbbVie, USA) on a heating/ECG pad (Fujifilm, Toronto, Canada). The heart rate was kept above 400/min. Loops were recorded with the VEVO 3100 micro-ultrasound imaging system (Fujifilm, Canada) and the left ventricular (LV) ejection fraction, stroke volume, cardiac output, left ventricular end-systolic volume (LVESV) and left ventricular end-diastolic volume (LVEDV) were measured in the parasternal long axis with Vevo lab software (Fujifilm, Canada).

After euthanasia and preparation of the heart, the atrium was punctured and mice were perfused with 10 ml of ice-cold saline solution, followed by perfusion with 4% paraformaldehyde (PFA) (Sigma-Aldrich, USA). The heart was removed and placed in 4% PFA for 1 h, followed by washing in PBS. The heart was then incubated for 4 h in 10%, 20% and 30% sucrose solution (Sigma-Aldrich, USA), respectively, followed by placement in optimal cutting temperature compound (OCT, Sakura Tissue Tek, The Netherlands) and cryosectioning. Histological analysis of murine hearts was performed with Masson’s trichrome (fibrosis staining) and haematoxylin–eosin according to standard protocols. Immunohistochemistry was performed using an anti-CD68 monoclonal antibody (FA-11, 50 µl per slide, 1:500 dilution; 30 min, RT; Bio-Rad, USA) or an isotype control (Invitrogen, USA). Slides were then incubated with a secondary anti-rat-biotin-conjugated antibody (50 µl per slide, 1:200, 30 min, RT; Vector Labs, United Kingdom). The Vectastain® ABC Kit components (containing Avidin-HRP) were then added according to the manufacturer’s instructions (Vector Labs, United Kingdom). Images were acquired using an AxioImager M2 microscope and Zen Black 2.3 software (both: Zeiss, Germany) for image analysis.

### Assessment of monocyte activation in ECMO and STEMI patients

Citrated whole blood was slowly drawn from an arterial line of patients receiving extra-corporeal membrane oxygenation (ECMO) and immediately transported to the laboratory for analysis. ECMO was initiated due to either respiratory or circulatory failure, the most common indications being acute respiratory distress syndrome or severe cardiogenic shock. Patients were aged over 18 years and had no haematological malignancies. Blood was taken 12 h to 7 days after ECMO implantation.

In a separate study, citrated whole blood was slowly drawn from patients diagnosed with ST-elevation myocardial infarction (STEMI) by antecubital vein puncture at a single time point within 36 h of percutaneous coronary intervention (PCI). Blood was immediately transferred to the laboratory. Only patients who had received coronary stents for coronary thrombosis and vessel occlusion as determined by coronary angiography were included. Patients with NSTEMI or unstable angina were not enrolled in this study. Patients with haematological malignancies or acute bacterial and viral infections were excluded.

Patient characteristics of ECMO and STEMI patients were taken from the electronic patient data management system and are presented in Online Resource 4 and 5. Citrated whole blood from healthy consenting volunteers with no known comorbidities or medication during the last 14 days was obtained for control experiments.

Using flow cytometry, binding of F7 to PMA-stimulated and unstimulated CD14^+^ monocytes from lysed whole blood was quantified using the appropriate controls. Whole blood was prepared as described for human samples under Flow-cytometric binding assays. Percentage of binding was calculated by dividing the mean fluorescence intensity of unstimulated samples by the MFI of PMA-stimulated samples, to account for lower stimulability in patients compared to healthy controls.

### Study approval and registration

Experiments involving human or animal samples were approved by the institutional review board of the University of Freiburg, Germany, and the Alfred Hospital, Melbourne, Australia. Participants or their legal representatives (in the case of ECMO patients) gave written informed consent in accordance with the Declaration of Helsinki. The cross-sectional observational studies were registered in the German Clinical Trials Register: DRKS00005277 (STEMI-patients), DRKS00011106 (ECMO patients).

### Statistics

Data are presented as mean ± SEM and analysed using two-tailed Student’s *t*-test when two groups were compared or one-way ANOVA with a Tukey post-hoc test when three or more groups were compared. Experiments were performed at least three times and were measurements from distinct biological replicates unless stated otherwise. Statistics were performed using GraphPad Prism 8.0 software (GraphPad, USA). *p* < 0.05 was considered statistically significant.

## Results

### Biological activity of recombinant mouse I-domain

Panning was performed on the recombinant mouse α_M_ I-domain, which has a sequence identity of 80% with the human α_M_ I-domain (according to protein sequence alignment by https://blast.ncbi.nlm.nih.gov/ based on the Swissprot library). The rationale was to select a DARPin which could be tested in mouse preclinical models. There was a high likelihood of selecting a DARPin which would display cross-reactivity to the human α_M_ I-domain due to the large sequence identity. To demonstrate that the recombinantly produced mouse I-domain, which is used as a target epitope for phage display, is biologically active, murine ICAM-1 was coated on 96-well plates. Recombinant mouse α_M_ I-domain binds to murine ICAM-1 in comparison to BSA-coated wells (Fig. 1b).

### ***Anti-α***_***M***_*** I-domain phage panning***

After three panning rounds on recombinant mouse α_M_ I-domain, specific enrichment of anti-α_M_ I-domain DARPin phages was found (Fig. 1c). DARPin-encoding plasmid inserts were cloned into the expression vector pMPAG-6 and several hundred DARPin clones were screened for their binding to the α_M_ I-domain. Of these DARPin clones, the clone F7 (= “DARPin F7”) was found to bind most specifically to the recombinant α_M_ I-domain (Fig. 1d). Homology analysis of selected clones showed that three different “families” of anti-mId DARPin clones were selected within the three panning rounds, indicating a highly efficient panning strategy (Online Resource 6).

### Expression and purification of DARPins in *E. coli* and endotoxin removal

DARPins were successfully expressed and purified with high yields (concentration of DARPins was up to 5 mg/ml) (Fig. 1e). Before endotoxin elimination, DARPin samples contained approximately 3 EU/ml (endotoxin unit/ml, 1 EU/ml corresponds to 0.1 ng endotoxin per ml). After endotoxin-removal (see “Methods” section), endotoxin concentration was below 0.1 EU/ml.

### ***Binding of DARPin F7 to mouse α***_***M***_*** I-domain and mouse monocytes***

**Fig. 2:**
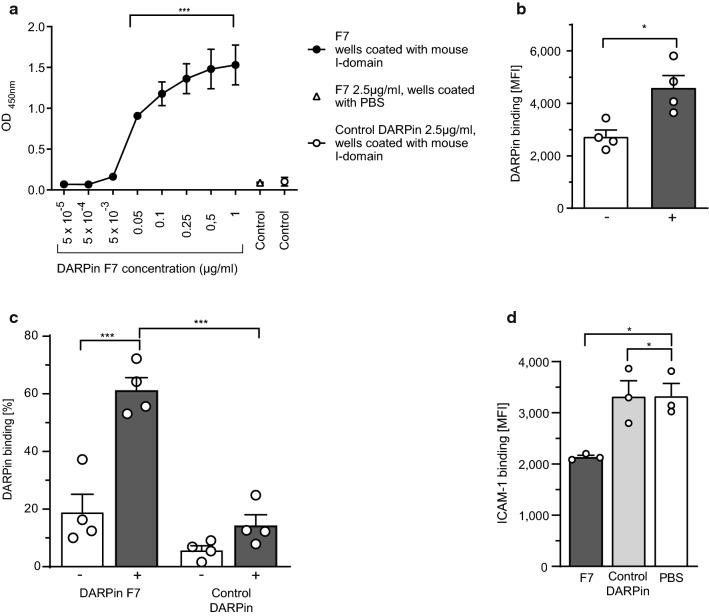
Characterizing DARPin F7**.** **a** Increasing concentrations of F7 led to increased signals at OD 450 nm in ELISA, indicating concentration-dependent binding behaviour. The *x*-axis shows increasing concentrations of DARPin F7. Controls are also shown. Wells were incubated overnight at 4 °C with recombinant mouse α_M_ I-domain. As negative controls, PBS and the control DARPin were used. F7 binding was detected via Streptavidin HRP using OD measurements at 450 nm (*n* = 3, ***p < 0.001). *p*-values were calculated by one-way ANOVA. **b** RAW 264.7 cells were cultured according to standard protocols. 5 × 10^5^ cells were resuspended in 100 µl PBS + Ca^2+^ and Mg^2+^ and stimulated with phorbol 12-myristate 13-acetate (PMA). PMA was added to stimulate cells and induce Mac-1 conformational change towards the activated conformation on the cellular surface. Cells were then stained with DARPins (*F*_c_ = 2.5 µg/ml). RAW 264.7 cells were identified based on scatter gating and their CD11b expression using a FITC anti-CD11b antibody. DARPin binding was detected using an Alexa Fluor 488 anti-His-tag antibody. Flow-cytometric analysis showed that F7 bound significantly stronger to PMA-simulated “+ ” compared to unstimulated “−” RAW 264.7 cells (*n* = 4, *p < 0.05). *p*-value was calculated by an unpaired two-tailed Student’s *t*-test. **c** 100 µl of mouse blood was stimulated with PMA (*F*_c_ = 200 ng/ml, 15 min, 37 °C) followed by red blood cell lysis. PMA was added to stimulate monocytes and induce Mac-1 conformational change towards the activated conformation on the monocyte surface. Cells were stained with a Pacific Blue anti-Ly-6C antibody and DARPins (*F*_c_ = 2.5 µg/ml). Binding of DARPin F7 to Ly-6C^++^ monocytes (defined as the top 3% percent of Ly-6C positive cells) is shown. Location of the monocytes in the monocyte gate was verified by backgating. DARPin binding was detected using an Alexa Fluor 488 anti-His-tag antibody. For more information, please see our gating strategy (Online Resource 1). No relevant binding of F7 to unstimulated “−” Ly-6C^++^ mouse blood monocytes was found. However, F7 bound significantly more to PMA-stimulated “+ ” monocytes. The control DARPin did not bind significantly to non-activated or activated Ly-6C^++^ mouse monocytes (*n* = 4, ***p < 0.001). **d** Monocytes were isolated from healthy C57BL/6N mice using a monocyte isolation kit (STEMCELL, France), stimulated with PMA (10 ng/ml, 15 min, 37 °C) and stained with DARPins (5 µg/ml, 15 min, RT) followed by incubation with rICAM (15 min, RT). ICAM-1-binding was detected by a FITC anti-ICAM-1 antibody (Biolegend, USA). Isolated monocytes were identified by forward vs side scatter gating. Competitive binding of F7 vs fluorescent recombinant ICAM-1 (rICAM) revealed that F7 significantly reduced binding of ICAM-1 to primary mouse monocytes (*n* = 3, *p < 0.05). *p*-values were calculated by one-way ANOVA. The error bars indicate SEM

F7 showed concentration-dependent specific binding to recombinant mouse α_M_ I-domain (Fig. 2a). Phorbol 12-myristate 13-acetate (PMA) was used to activate monocytes resulting in increased activation of Mac-1 [25]. F7 bound significantly stronger to PMA-stimulated than to unstimulated (immortalized) mouse monocytes (RAW 264.7) (Fig. 2b). Specific binding of F7 in comparison to a control DARPin (DARPin E3_5) [32] to activated monocytes was confirmed for Ly-6C^++^ mouse blood monocytes (Fig. 2c).

### DARPin F7 reduces monocyte–ICAM-1 interaction in flow cytometry

The capacity to block ICAM-1–Mac-1 interaction was demonstrated by flow cytometry (Fig. 2d). Pre-incubation of mouse monocytes with F7 led to significantly reduced binding of recombinant ICAM-1 compared to treatment with the control DARPin or PBS.

### ***Docking model and mutational analysis of DARPin F7 and mouse α***_***M***_*** I-domain***

**Fig. 3:**
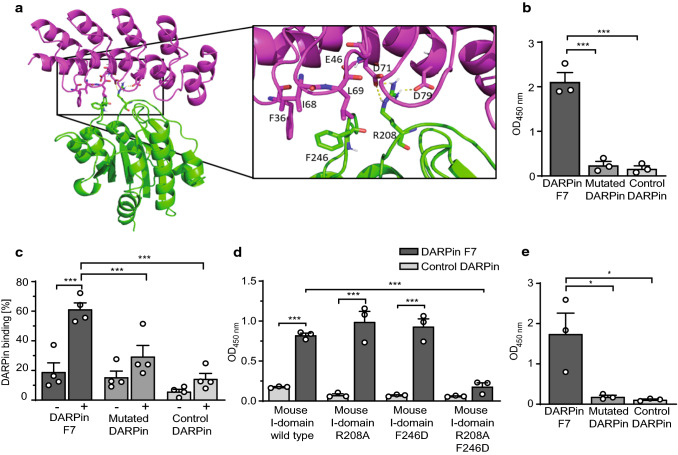
Defining specific interaction residues of DARPin F7 to mouse α_M_ I-domain using homology modelling and docking studies. **a** Model of F7 (*magenta*) interacting with the mouse α_M_ I-domain (*green*). Shown via *magenta* sticks are the three F7 residues (E46, D71, D79) proposed to be important for binding via polar interactions and the three F7 residues (F36, I68, L69) proposed to be involved in hydrophobic interactions. Shown via *green* sticks are the two mouse α_M_ I-domain residues (R208, F246) proposed to be important for binding. Also shown via *yellow dashed lines* are the putative hydrogen bonds. (**b**) Wells were coated with mouse α_M_ I-domain and biotinylated DARPins (*F*_c_ = 1 µg/ml) were added. After several washing steps, Streptavidin-HRP was added, followed by TMB-substrate and the OD_450nm_ was quantified in an ELISA plate reader. The F7 alanine triple mutant (E46A_D71A_D79A, “mutated DARPin”) bound significantly less to the mouse α_M_ I-domain compared to F7. (*n* = 3, ***p < 0.001). **c** 100 µl of mouse blood was stimulated with PMA (*F*_c_ = 200 ng/ml, 15 min, 37 °C) followed by red blood cell lysis. PMA was added to activate monocytes and induce Mac-1 conformational change towards the activated conformation on the monocyte surface. Cells were stained with a Pacific Blue anti-Ly-6C antibody and DARPins. Binding of DARPin F7 to Ly-6C^++^ monocytes (defined as the top 3% percent of Ly-6C positive cells) is shown. Location of the monocytes in the monocyte gate was verified by backgating. DARPin binding was detected using an Alexa Fluor 488 anti-His-tag antibody. Flow-cytometric analysis showed that F7 bound significantly more strongly to PMA-stimulated “ + ” compared to unstimulated "−" mouse monocytes. Also, F7 bound significantly more to stimulated mouse monocytes compared to mutated and control DARPin. This indicates that the residues (E46, D71, D79) contribute to F7 binding to mouse Ly-6C^++^ monocytes. There is a small degree of unspecific background binding of the mutant DARPin in our flow cytometric whole blood assay (*n* = 4, ***p < 0.001). **d** For this ELISA, wells were coated with mouse α_M_ I-domain or mouse α_M_ I-domain mutants and biotinylated DARPins (*F*_c_ = 1 µg/ml) were added. After several washing steps, Streptavidin-HRP was added, followed by TMB-substrate and the OD_450nm_ was quantified in an ELISA plate reader. Binding of F7 to the mouse α_M_ I-domain double mutant F246D_R208A was significantly reduced compared to the mouse α_M_ I-domain wild type (*n* = 3, ***p < 0.05). F246 and R208 therefore most likely constitute the epitope of F7 on the mouse α_M_ I-domain. **e** ELISA was performed as described in **d**. Binding of F7 was not affected by the conformational shift induced by the activating F302L mutation in the mouse α_M_ I-domain (*n* = 3, *p < 0.05). *p*-values were calculated by one-way ANOVA. The error bars indicate SEM

Homology modelling and computational docking were performed to identify the interaction sites between F7 and the mouse α_M_ I-domain. Two potential protein–protein models were generated. In both models, three F7 residues (E46, D71, D79) were predicted to be important for the interaction with the mouse α_M_ I-domain. In the first model (not shown), the F7 side chain E46 was predicted to hydrogen-bond with the side chain of the mouse α_M_ I-domain N206, D71 with the side chain of the mouse α_M_ I-domain N146, and D79 with the backbone of mouse α_M_ I-domain I148. There was also a predicted salt bridge between the side-chains of F7 R13 and the mouse α_M_ I-domain E179. In the second model, mouse α_M_ I-domain R208 was predicted to be involved in a bifurcated salt bridge and hydrogen-bond network with F7 D71 and D79 (Fig. 3a). Mouse α_M_ I-domain F246 was predicted to be involved in hydrophobic interactions with F7 F36, I68, and L69 (Fig. 3a). To biologically assess these models, mutagenesis of F7 and mouse α_M_ I-domain was conducted and their ability to still interact was assessed.

An alanine triple mutant of F7 (E46A_D71A_D79A = mutated DARPin, mF7) was created and did not bind to the mouse α_M_ I-domain (Fig. 3b). Furthermore, the mutated DARPin showed significantly reduced binding to activated mouse monocytes compared to F7 in mouse Ly-6C^++^ monocytes (Fig. 3c).

For the mouse α_M_ I-domain, the following mId-mutants were created and assessed: N146A_E179A_N206A, R208A, F246D, R208A_F246D, N146A, E179A, N206A. The N146A_E179A_N206A did not result in loss of binding to F7 (data not shown); however, the double mutation R208A_F246D of the mouse α_M_ I-domain did not show significant binding to F7 (Fig. 3d), whereas the mouse α_M_ I-domain single mutations (R208A, F246D) alone were not able to alter the binding affinity to F7 (Fig. 3d). In summary these data support the second model as depicted in Fig. 3a.

An overview of mutation sites located within the sequences of DARPin F7 or the mouse α_M_ I-domain are presented in Online Resource 2 and 3.

It has been postulated that the α_M_ I-domain itself undergoes significant conformational changes upon integrin activation beyond the exposure of the I-domain as a consequence of Mac-1 activation. The active conformation can be induced by the F302L mutation, creating a constitutively activated mouse α_M_ I-domain [36]. Binding of F7, mF7, and the control DARPin to mId_F302L was similar to the wild-type mId, indicating that the epitope within the α_M_ I-domain to which F7 binds is not affected by conformational changes in the α_M_ I-domain (Fig. 3e).

### *DARPin F7 inhibits monocyte–endothelial interaction *in vivo

**Fig. 4:**
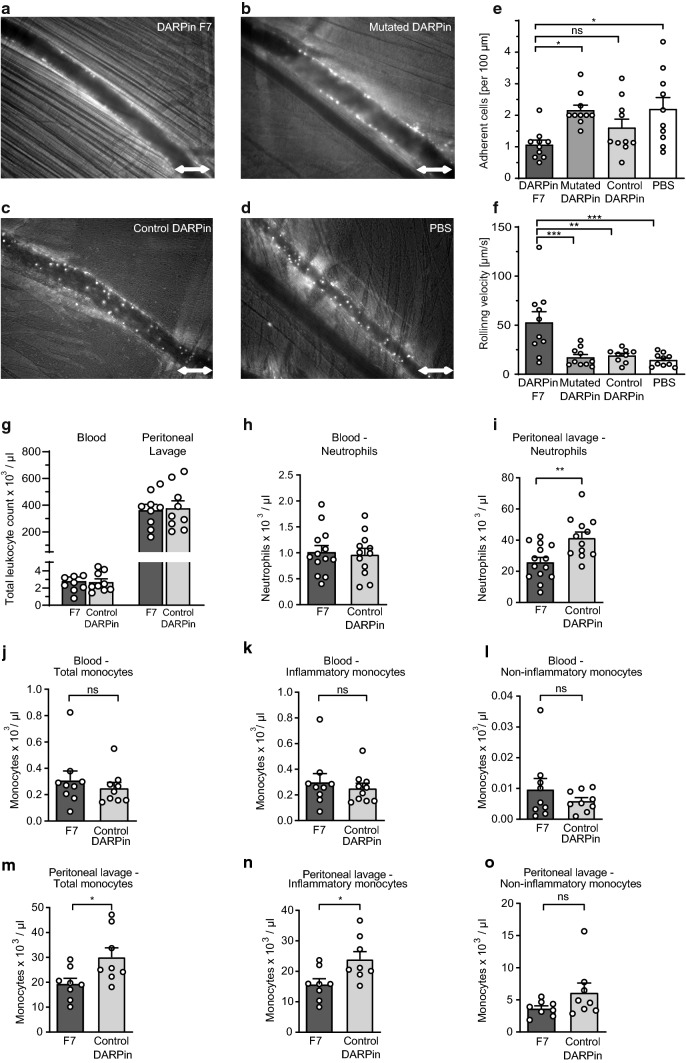
DARPin F7 inhibits monocyte–endothelial interaction in vivo. Representative images of mice treated with **a** DARPin F7, **b** mutated DARPin, **c** control DARPin, or **d** PBS taken during intravital microscopy in a cremaster mouse model. Scale bar = 100 µm. Inflammation was triggered by 200 ng recombinant mouse TNFα (ThermoFisher Scientific, USA*)* i.p. 3 h before surgery. Cells were labelled by 50 µl rhodamine 6G injection (0.1% solution) after anesthesia. 100 µg DARPin or an equivalent volume of PBS were injected into the femoral vein, and microscopy and recordings were performed immediately. The number of rolling leukocytes and the number of adherent leukocytes per 100 µm vessel wall were quantified. Leukocytes not moving for more than 30 s were considered adherent. Rolling velocity was calculated by dividing the distance (100 µm) by the time (in seconds) for different leukocytes to travel 100 µm through the vessel (in µm/s). **e** The number of adherent cells in mice treated with F7 was significantly reduced compared to controls (*n* = 10 animals in each treatment group, *p < 0.05, ns = not significant). **f** Also, the rolling velocity was significantly increased in mice treated with F7 compared to those treated with controls (*n* = 10 animals in each treatment group, **p < 0.01, ***p < 0.001). *p*-values were calculated by one-way ANOVA. **g**–**o** In a CLP mouse model of sepsis, mice received 200 µg of DARPins 1 h before surgery by tail-vein injection. Mice were anaesthetised followed by surgical opening of the peritoneum, location, ligation and puncture of the cecum. After surgery, mice were monitored and euthanized 20 h after surgery when blood and the peritoneal lavage (PL) were obtained and flow cytometry was performed. Total leukocyte counts were determined by an automated cell counter. Leukocyte subsets were counted by flow cytometry. Total monocytes were identified as CD45^+^CD11b^+^CD115^+^. From this population inflammatory (CD45^+^CD11b^+^CD115^+^Ly-6G (Gr-1)^+^) or non-inflammatory (CD45^+^CD11b^+^CD115^+^Ly-6G(Gr-1)^−^) monocytes were differentiated. Neutrophils were identified as CD45^+^CD11b^+^Ly-6G(Gr-1)^+^CD115^−^. **g** Total leukocyte counts in whole blood or peritoneal lavage of septic mice treated with F7 or the control DARPin did not significantly differ for either treatment. **h** Numbers of neutrophils in blood were similar in both groups. **i** Significantly reduced numbers of neutrophils were observed in the peritoneal lavage in mice treated with F7. **j**–**l** Numbers of total monocytes, inflammatory and non-inflammatory monocytes in peripheral blood were not significantly different between both treatments. **m, n** However, significantly reduced monocyte and inflammatory monocyte counts were observed in the peritoneal lavage (PL) of mice treated with F7, indicating inhibition of monocyte migration into the peritoneum by F7. **o** Numbers of non-inflammatory monocytes in the peritoneal lavage of mice treated with F7 were lower, although significance was not achieved. *n* = 9–12 mice per treatment group, *p < 0.05, **p < 0.01, *p*-values were calculated by an unpaired two-tailed Student’s *t*-test. The error bars indicate SEM

To investigate the in vivo effects of F7 on leukocyte function, we conducted intravital microscopy using the cremaster mouse model. We were able to show that mice treated with F7 had reduced numbers of adherent leukocytes visualized by fluorescence microscopy using rhodamine staining (Fig. 4a–e). Furthermore, the rolling velocity of leukocytes in mice treated with F7 was significantly increased, indicating an inhibitory effect of F7 on Mac-1 function in vivo (Fig. 4f).

### Anti-inflammatory effect of DARPin F7 in CLP model

Inhibition of leukocyte migration by activation-specific Mac-1 blocking was further assessed in a mouse sepsis CLP model. Mice treated with either F7 or the control DARPin had similar total leukocyte counts in blood or peritoneal lavage (Fig. 4g) as determined by automated cell counting. Using flow cytometry, we found similar levels of neutrophils in blood in mice treated with F7 or the control DARPin. However, significantly reduced levels of neutrophils were counted in the peritoneal lavage (Fig. 4h,i). Moreover, mice treated with F7 had similar numbers of total monocytes (CD45^+^CD11b^+^CD115^+^), inflammatory (CD45^+^CD11b^+^CD115^+^Ly-6G(Gr-1)^+^) and non-inflammatory (CD45^+^CD11b^+^CD115^+^Ly-6G(Gr-1)^−^) monocytes compared to the control DARPin in blood (Fig. 4j–l); however, mice treated with F7 had significantly reduced numbers of total and inflammatory monocytes in the peritoneal lavage (Fig. 4m–n). Additionally, we determined cytokine levels in blood and the peritoneal lavage from mice in both groups and found similar levels in both treatment groups (Online Resource 7). No differences in weight loss or survival between the different treatment groups were observed in the 20 h monitoring period after surgery until euthanasia.

### DARPin F7 prevents loss of left ventricular function in murine myocarditis

Myocarditis is an inflammatory cardiomyopathy that appears in approximately 1.5 million people worldwide annually [29]. Overshooting inflammatory reactions are seen as a major cause of myocardial damage in myocarditis [64]. Expression of ICAM-1 by cardiomyocytes could mediate interaction with monocytes expressing Mac-1, leading to potential cardiomyocyte dysfunction. We, therefore, investigated ICAM-1 expression in mouse cardiomyocytes and Mac-1 expression in mouse monocytes by qPCR, confirming significant expression of Mac-1 in isolated mouse monocytes and expression of ICAM-1 in mouse cardiomyocytes (relative mRNA expression of Mac-1: monocytes unstimulated vs stimulated: 1.00 ± 0.30 vs 2.38 ± 0.45; *n* = 5, *p* < 0.05; relative mRNA expression of ICAM-1 on mouse cardiomyocytes unstimulated vs stimulated: 1.00 ± 0.42 vs 2.84 ± 0.59, *n* = 5, *p* < 0.05, Online Resource 8). Endothelial cells, however, also highly express ICAM-1 [44].

**Fig. 5:**
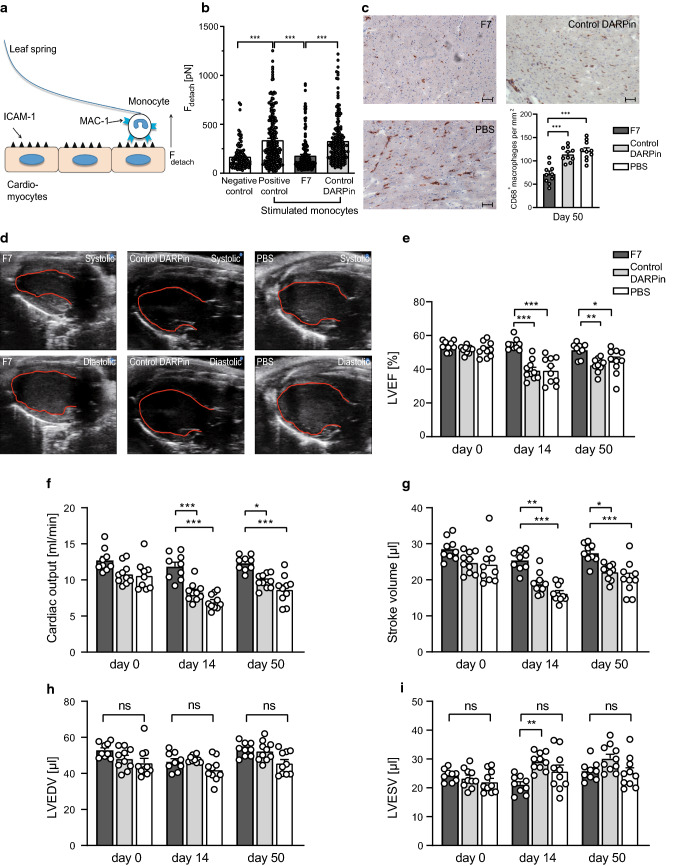
DARPin F7 improves LV function in a murine model of myocarditis by inhibition of monocyte–cardiomyocyte interaction. **a** Schematic representation of how atomic force microscopy based SCFS was performed. Detachment force (*F*_detach_) was assessed by repeated removal of monocytes adhering to cardiomyocytes using a leaf spring. Mouse cardiomyocytes used in SCFS were isolated from whole murine hearts. After removal, the murine hearts were connected to a Langendorff perfusion system and perfused with collagenase containing buffers up to 50 min and isolated cardiomyocytes were then cultured in laminin-coated cell-culture dishes. Mouse monocytes were isolated from healthy C57BL/6N mice using a monocyte isolation kit, stimulated with PMA to activate cells and induce Mac-1 conformational change towards the activated conformation. Afterwards, monocytes were treated with DARPins. The negative control was not stimulated with PMA, the positive control was stimulated with PMA but was not treated with DARPins. Cardiomyocytes were stimulated with mouse TNFα before SCFS (**b**) SCFS revealed significantly reduced *F*_detach_ for stimulated monocytes and cardiomyocytes pre-incubated with F7 compared to the control DARPin (*n* = total number of measurements of *F*_detach_: negative control *n* = 97, positive control *n* = 226, F7 *n* = 167, Control DARPin *n* = 162; data from at least five monocyte–cardiomyocyte pairs per treatment group. We aimed for at least 20 measurements of *F*_detach_ from each pair, ***p < 0.001). **c** Immunohistochemistry was performed using an anti-CD68 monoclonal antibody (FA-11) or an isotype control followed by incubation with a secondary anti-rat-biotin-conjugated antibody. The Vectastain^®^ ABC Kit components (containing Avidin-HRP) were then added according to the manufacturer’s instructions. Images were acquired using an AxioImager M2 microscope and analysed using Zen Black 2.3 software. Significantly reduced numbers of macrophages (*brown colour*) were found for mice treated with F7 compared to treatment with the control DARPin and PBS (*n* = 10 mice per treatment group, ***p < 0.001). *p*-values were calculated by one-way ANOVA, Scale bar = 50 µm. **d, e** Echocardiography was performed under isoflurane anesthesia on a heating/ECG pad. The heart rate was kept above 400/min. The left ventricular (LV) ejection fraction was measured in the parasternal long axis. Echocardiography revealed that mice treated with F7 did not suffer significant reduction in left ventricular ejection fraction (LVEF) on day 14 and day 50 compared to mice treated with the control DARPin or PBS in a murine model of myocarditis. Similar observations were made regarding cardiac output (**f**) and stroke volume (**g**). No differences between treatment groups were observed regarding the left ventricular enddiastolic volume (LVEDV) (**h**) and left ventricular endsystolic volume (LVESV) (**i**). Examples of echocardiographic loops from representative mice are provided online as Online Resource 13–15 (*n* = 9 mice per treatment group, ***p < 0.001). *p*-values were calculated using one-way ANOVA. The error bars indicate SEM

To investigate whether monocytes attach to cardiomyocytes in an α_M_ I-domain-dependent manner with subsequent cardiomyocyte dysfunction, atomic force microscopy (AFM) based single-cell force spectroscopy (SCFS) was performed (Fig. 5a). The force necessary to detach stimulated mouse monocytes which had been pre-incubated with F7 from mouse cardiomyocytes was significantly reduced compared to the control DARPin or PBS, indicating an inhibiting effect of F7 (Fig. 5b). These data indicate that the interaction of monocytes with cardiomyocytes can be inhibited by F7. We then assessed the therapeutic effect of F7 on acute, myosin-induced autoimmune myocarditis using a well-described murine model [46]. After induction of myocarditis, mice did not show severe signs of distress or lose weight. A significant influx of monocytes and neutrophils into the myocardium on day 14 was observed using flow cytometry of lysed mouse hearts indicating a successful induction of myocarditis (Online Resource 9). At day 50 after euthanasia, histology was performed. The number of macrophages (CD68^+^) was significantly reduced in animals treated with F7 compared to controls (Fig. 5c). Interestingly, in the staining obtained, monocytes/ macrophages seemed to be localized within the myocardium. As expected, Masson’s trichrome staining revealed no signs of fibrosis on day 50 with no obvious differences between the three treatment groups (Online Resource 10). Additionally, haematoxylin–eosin staining did not reveal relevant inflammatory infiltrates on day 50 (chronic phase), also as expected (Online Resource 11). Moreover, cytokine levels of IFNβ, IL-1a and MCP-1 in plasma were investigated on day 14. Low levels were found with no significant differences between treatment groups (Online Resource 12).

The effect of F7 on LV function after induction of myocarditis was investigated via echocardiography (Fig. 5d, e). At day 0 (the time point of myocarditis induction), no differences in left ventricular ejection fraction, cardiac output and stroke volume were found between the three groups. On day 14 and day 50 after the first induction of an immune response, in the acute and chronic phase of myocarditis, these parameters differed significantly between the treatment groups and were best in the F7 group (Fig. 5e–g). Left ventricular enddiastolic volume (LVEDV) and left ventricular endsystolic volume (LVESV) showed no significant differences between treatment groups (Fig. 5h, i). Examples of echocardiographic loops on day 14 from representative mice are provided as Online Resources 13–15.

### DARPin F7 reduces inflammation in myocardial infarction

An overshooting inflammatory reaction is one of the major drivers of ischaemia/reperfusion (I/R) injury after myocardial infarction [35] and anti-inflammatory therapy has been shown to preserve cardiac function [84]. Targeting activated Mac-1 may, therefore, be a novel therapeutic option to possibly reduce overshooting inflammation causing cardiac ischaemia/reperfusion injury. Using a mouse model of cardiac ischaemia/reperfusion injury (30 min LAD ligation), we found significantly reduced levels of CD68^+^ macrophages and CD8^+^ cells in the myocardium of mice treated with F7 compared to the control DARPin. Furthermore, a reduced infarct size was observed histologically in mice treated with F7 (Online Resource 16).

### Translation of DARPin F7 binding to human leukocytes

**Fig. 6:**
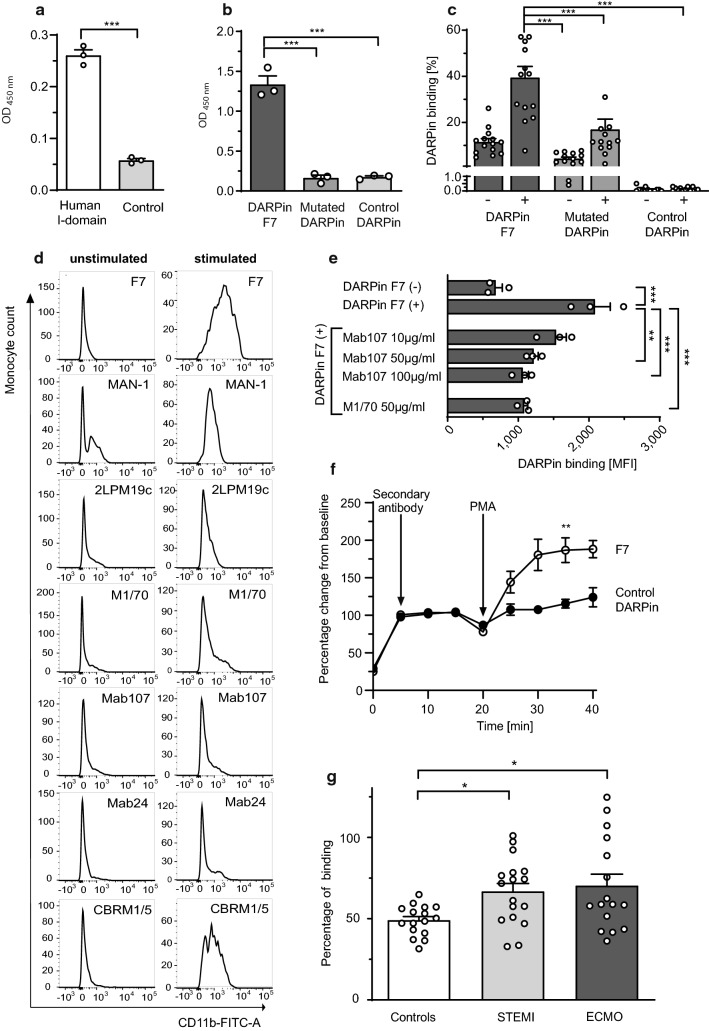
Translation of DARPin F7 to human applications. **a** In this ELISA, wells were coated with recombinant human ICAM-1. The recombinant human α_M_ I-domain or BSA was added to the wells and binding was detected by an anti-His-HRP antibody and quantified at OD_450nm_ in an ELISA plate reader. It was found that recombinant ICAM-1 bound significantly more to human α_M_ I-domain than to the control (BSA) indicating functionality of the human α_M_ I-domain (*n* = 3, ***p < 0.001). *p*-value was calculated by an unpaired two-tailed Student’s *t*-test. **b** For this ELISA, wells were coated with the recombinant human α_M_ I-domain and biotinylated DARPins were added (*F*_c_ = 1 µg/ml). DARPin binding was detected by a Streptavidin-HRP antibody and quantified at OD_450nm_. DARPin F7 bound significantly more to the immobilized human α_M_ I-domain compared to the control DARPin or the mutated DARPin in ELISA (*n* = 3, ***p < 0.001). **c** Human whole blood monocytes were stimulated with PMA (stimulated, “ + ”) or PBS (unstimulated, “-”). PMA was added to activate cells and induce Mac-1 conformational change on the monocyte surface. Staining and gating for classical monocytes (CD14^+^) was performed as described in Online Resource 1. DARPin binding was detected by a secondary Alexa Fluor 488 anti-His-tag antibody. F7 bound significantly more to stimulated “ + ” human CD14^+^ monocytes compared to unstimulated “-” monocytes. F7 also bound stronger to stimulated “ + ” monocytes than the mutated DARPin and the control DARPin. There is a small degree of unspecific background binding of the mutant DARPin in our flow cytometric whole blood assay (*n* = 14, ***p < 0.001, *p*-values were calculated by one-way ANOVA). **d** The same flow cytometric assay as in **c** was carried out. The detection of the population shift occurring after CD14^+^ monocyte stimulation with PMA was compared between DARPin F7 (2.5 µg/ml) and other anti-Mac-1 antibodies M1/70, Mab24, MAN-1, Mab107, CBRM1/5, and 2LPM19c (all 10 µg/ml). Visual analysis shows a larger population shift detected by DARPin F7 compared to the other anti-Mac-1 antibodies. It was concluded that due to this capacity, F7 binds conformation-specifically to activated Mac-1 on PMA-stimulated, activated human monocytes (representative histograms of *n* = 3). **e** The flow cytometric assay was conducted as described for Fig. 3c. However, before the addition of DARPins, stimulated “ + ” human whole blood monocytes were pre-incubated with anti-CD11b antibodies Mab107 and M1/70. F7 binding to activated monocytes was reduced by competition with anti-CD11b M1/70 and Mab107 at 50 µg/ml (*n* = 3, **p < 0.01, ***p < 0.001). *p*-values were calculated by one-way ANOVA. According to our in-silico model Mab107 binding sterically mirrors binding of F7 to the α_M_ I-domain. *p*-values were calculated by one-way ANOVA. **f** Neutrophils were isolated from whole human blood using Polymorphprep©. 5 × 10^6^ neutrophils were stained with a Pacific Blue anti-CD66b antibody for identification. After 2 min of acquisition and recording, F7 or the control DARPin and the secondary Alexa Fluor 488 anti-His-Tag antibody were added. Recording was continued for a total of 20 min when PMA was added to stimulate neutrophils and induce Mac-1 conformational change towards the activated conformation on the neutrophil surface. Recording was continued for another 20 min and binding was detected in the FITC channel and is presented as percentage change from baseline since baseline binding between F7 and the control DARPin were different. F7 also bound significantly more to PMA-stimulated neutrophils compared to the control DARPin (*n* = 3, **p < 0.01 at 35 min, F7 vs control DARPin). *p*-value was calculated by an unpaired two-tailed Student’s *t*-test. **g** Observational patient studies were conducted to evaluate the capacity of F7 to detect monocyte activation in patients. Citrated blood was drawn from ECMO or STEMI patients and F7 binding to CD14^+^ monocytes was determined by flow cytometry as previously described in **c** and in the “Methods” section. F7 bound significantly more to CD14^+^ monocytes of ECMO and STEMI patients than to healthy controls, indicating increased monocyte activation in these patients (ECMO *n* = 16 patients, STEMI *n* = 17 patients, healthy controls *n* = 16, *p < 0.05). *p*-values were calculated by one-way ANOVA. The error bars indicate SEM

To provide proof of the translational relevance of our findings and ultimately the suitability of F7 for diagnostic and therapeutic applications in humans, we systematically tested F7 targeting human Mac-1. To ensure the functionality of our tools, we first demonstrated that the recombinant human α_M_ I-domain significantly bound to its ligand, human ICAM-1 (Fig. 6a). Using this human α_M_ I-domain, we demonstrated that F7 binds specifically to the human α_M_ I-domain compared to the controls, mutated DARPin and non-binding control DARPin (Fig. 6b). This confirms the cross-reactivity between mouse and human Mac-1.

Next, we determined whether F7 binds to activated human monocytes. Whereas the control DARPin does not bind to human monocytes at all, the mutated F7 shows some binding to human monocytes, and F7 shows strong binding to PMA-activated monocytes (Fig. 6c). We then compared F7 with known anti-Mac-1 antibodies. An increased level of binding to PMA-activated monocytes in flow cytometry was observed for F7 compared to conventional anti-CD11b antibodies 2LPM19c, M1/70, Mab107, CBRM1/5, Mab24, and the anti-Mac-1 scFv MAN-1. Visual analysis of the respective histograms shows a larger population shift for F7 when comparing unstimulated and stimulated samples (Fig. 6d), indicating stronger binding to activated monocytes. Selective binding to Mac-1 was further confirmed by direct competition of binding of F7 by anti-Mac-1 antibodies with defined epitopes. F7 binding to PMA-stimulated CD14^+^ monocytes was significantly reduced if monocytes were pre-incubated with anti-Mac-1 antibodies M1/70 or Mab107, further supporting a conformation-specific mode of binding of F7 to activated monocytes (Fig. 6e).

As neutrophils in addition to monocytes are important targets for anti-inflammatory therapy, we assessed the binding of DARPins to Mac-1 on neutrophils. F7 binding to neutrophils was investigated in a time-dependent flow-cytometric assay. F7 binding significantly increased compared to the control DARPin after PMA stimulation of neutrophils (Fig. 6f).

### Binding of DARPin F7 on monocytes in patients with ECMO, sepsis and STEMI

To provide proof-of-concept data for the suitability of F7 to detect monocyte activation in a clinical setting, we investigated patients with three conditions in which monocyte activation has been proposed: ECMO, sepsis and STEMI. Significantly increased binding of F7 to CD14^+^ monocytes was observed in whole blood from ECMO patients compared to healthy controls (Fig. 6g). In a cohort of patients with pulmonary sepsis, monocyte activation was detected as well (Online Resource 17).

Also, F7 bound significantly stronger to CD14^+^ monocytes from STEMI patients compared to healthy controls (Fig. 6g). An overview of patient characteristics is provided online (Online Resource 4 and 5).

## Discussion

The aim of the present study was to select and test DARPins that preferentially bind to the activated conformation of the leukocyte integrin Mac-1 in order to obtain, first, a diagnostic tool for the assessment of monocyte activation and, second, a therapeutic anti-inflammatory agent selectively targeting activated monocytes.

By choosing the α_M_ I-domain of murine Mac-1, which shares a high sequence homology with the α_M_ I-domain of human Mac-1, as a target for our DARPin selection, we generated and selected a DARPin that binds to mouse and human Mac-1. This cross-reactivity provided the opportunity to test the selected DARPin in therapeutic approaches in preclinical mouse disease models and thus to greatly facilitate a future drug development programme in humans.

Different strategies have been employed to generate activation-specific antibodies to Mac-1. Diamond et al. selected immunoglobulins from hybridoma supernatants for their ability to bind PMA-activated neutrophils, while Eisenhardt et al. employed a subtractive panning strategy using human scFv phage libraries on Mac-1-expressing CHO cells [20, 24]. Both were cell-based selection strategies, whereas our approach is based on the use of immobilized recombinant mouse α_M_ I-domain to select activation-specific binders from a DARPin phage library (Fig. 1). Our strategy is supported by the fact that the epitopes of MAN-1 and CBRM1/5 are also located on the α_M_ I-domain of Mac-1 [12, 21]. It, therefore, resembles physiological ligand binding to the activated receptor conformation [6]. Competitive orthosteric activation-specific blocking of Mac-1 by F7, which prevents ligand interaction, may offer several advantages over an allosteric binding mode, which can induce an activated, pro-adhesive state of the integrin with severe immune activation and reduced outcomes [3].

The selected anti α_M_ I-domain DARPin F7 has significant advantages in comparison to immunoglobulins: it is relatively small (~ 17 kD) and thus can penetrate more easily into tissue, it is highly thermo- and pH-stable, is less likely to induce allergic reactions, and can be easily produced in bacteria at large scales [42]. F7 showed dose-dependent binding to the mouse α_M_ I-domain and specific binding behaviour to PMA-activated mouse monocytes compared to the control DARPin. Additionally, ICAM-1, which also binds to activated Mac-1 via the I-domain, was inhibited by F7, indicating competitive, specific binding of F7 [22].

To further characterize the binding of F7 to the α_M_ I-domain, a mutational analysis of F7 was conducted. We determined that the paratope on F7 is comprised of the residues E46, D71, and D79, since the F7 alanine triple mutant failed to bind to the human or mouse Mac-1 α_M_ I-domains and displayed significantly reduced binding to activated human and mouse monocytes. These residues are located on the antigen-binding surface of F7. Interestingly, according to our in silico models, these loops sterically mirror the loops of the heavy chain of mAb107 when binding to the human α_M_ I-domain [52]. In support of this, we could demonstrate that in a competitive manner, mAb107 is able to significantly reduce the binding of F7 to human monocytes in flow cytometry. These data further endorse our proposed model of interaction between F7 and the α_M_ I-domain.

Our proposed model of binding may well be an established form of the α_M_ I-domain interacting with ligands [52]. It was demonstrated that R208 and F246 of the mouse α_M_ I-domain constitute the binding epitope for F7, resulting in less binding of F7 to the α_M_ I-domain with mutations of these residues. Interestingly, these two residues are conserved in the human α_M_ I-domain, offering an explanation for the specific binding of F7 to the human α_M_ I-domain and human monocytes. Additionally, other Mac-1 ligands bind to the same residues on the α_M_ I-domain. Among these, R208 is known to mediate binding to NIF (neutrophil inhibitory factor). Both R208 and F246 are required for binding to iC3b [62]. Furthermore, an epitope which is composed of residues located relatively far apart on the primary structure is characteristic of and in line with the mosaic model of Mac-1 proposed by Ustinov et al. [76]. Binding of F7 to the mouse α_M_ I-domain was also shown to occur independent of the I-domain’s conformational status. These binding characteristics can be explained by the fact that R208 and F246, the epitope for F7 on the mouse α_M_ I-domain, are not subject to conformational change upon I-domain activation [52].

Our hypothesis of activation-specific binding to activated Mac-1 is empirically further supported by increased binding of F7 to PMA-stimulated monocytes compared to other anti-Mac-1 antibodies including mAb107, which has a similar epitope, and CBRM1/5, an antibody claimed to bind specifically to activated Mac-1 [21, 52]. Various conformationally sensitive DARPins have been selected against different receptors, demonstrating the particular suitability of the DARPin technology for targeting specific conformations in target epitopes, and thus the increased affinity of F7 for activated Mac-1 on monocytes may be favoured by inherent properties of DARPins such as sterical advantages or smaller size [54]. In summary, F7 binds specifically to the activated receptor conformation of the leukocyte integrin Mac-1 and may serve as a diagnostic tool indicating monocyte activation status.

One of the initial steps in inflammation is leukocyte rolling, adhesion to endothelial cells and transmigration into the subendothelial space [28]. Several studies have found that the leukocyte integrin Mac-1 is particularly involved in slow rolling, firm adhesion, and intravascular crawling of leukocytes [23, 71]. To investigate the therapeutic blocking effect of F7 on leukocyte–endothelial interaction, intravital microscopy using a cremaster mouse model was performed [80]. Our data are in line with the reported role of Mac-1, as we found that F7 significantly increases rolling velocity and inhibits adherence of activated leukocytes on the vascular endothelium. These data indicate that F7 effectively inhibits leukocyte–endothelial interaction and thereby the early stage of (vascular) inflammation. This led us to explore the therapeutic anti-inflammatory effects of F7 in mouse models of inflammatory diseases.

Sepsis and septic shock are serious conditions associated with overshooting inflammation and high mortality. Treating the overshooting inflammation in sepsis using immunomodulatory therapies in the clinical setting has proven to be difficult [14], possibly due to the use of unspecific anti-inflammatory strategies. Targeting specifically and only activated Mac-1 on monocytes and neutrophils is an innovative approach that could lead to an effective agent for the management of overshooting inflammation in sepsis and septic shock. The mouse model of cecal ligation and sepsis is a well-established model for the study of severe sepsis and was chosen to determine whether F7 inhibits monocyte and neutrophil migration into areas of inflammation [63]. Neutrophils and monocytes (total monocytes and inflammatory monocytes) in peritoneal lavage were significantly reduced in animals that received F7. Monocyte count in blood, however, was not significantly different for mice treated with F7 or the control, indicating that F7 might have avoided monocyte migration into the peritoneal lavage.

Myocarditis is one of the leading causes of sudden cardiac death and cardiac transplantation [17]. Although myocarditis is believed to be a T-cell driven disease, the pathological effects and myocardial damage are often mediated by effector cells: particularly monocytes [77]. After infection with cardiotropic viruses or exposure to cardiotoxic substances, cardiomyocytes undergo necrosis and cell lysis, with subsequent infiltration of lymphocytes and monocytes into the cardiac tissue, finally resulting in an autoimmune reaction with impaired cardiac function and heart failure [16]. However, to this date there is no established targeted anti-inflammatory therapy available and management focuses on managing the complications of myocarditis [13, 65]. Due to the strong involvement of monocytes/macrophages in myocarditis [1], targeting activated Mac-1 is an attractive therapeutic approach. We investigated whether F7 is a potential, sought-after, novel anti-inflammatory drug for the treatment of myocarditis. The results obtained with F7 are striking. Loss of LV function could be prevented. Furthermore, we showed that the recruitment of monocytes/macrophages was reduced significantly. Most interestingly, using AFM based SCFS we could show that inhibition of activated Mac-1 by F7 reduces the adhesion of activated monocytes to murine cardiomyocytes. This provides a new aspect of the pathology of myocarditis and also suggests a new path of therapeutic intervention.

Our findings are in line with those of Kolattukudy et al., who found that migration of monocytes into cardiac tissue resulted in myocarditis with depressed contractile function [43]. The importance of macrophage accumulation in the cardiac tissue on prognosis in myocarditis patients was investigated by Kindermann and colleagues [41]. By analysing human endomyocardial biopsies, they found that immunohistological signs of inflammation such as CD68-positive cells, but not the abundance of cardiotropic viruses, predicted the outcome of patients with myocarditis. Hence, prophylactic inhibition of monocyte/macrophage accumulation into the cardiac tissue, as performed in the present study by targeting the activated conformation of Mac-1, represents a promising therapeutic approach for the treatment of myocarditis patients.

These data demonstrating the therapeutic potential of F7 can also serve as a model for the treatment of other inflammatory conditions. These include arthritis [74], glomerulonephritis [73], bullous pemphigoid [47], experimental autoimmune encephalopathy [30], inflammatory bowel disease [58], and ischaemic kidney injury [19].

Targeting Mac-1 may also carry risks. Mac-1 deficiency is associated with an increased susceptibility to bacterial infection due to an impeded neutrophil response [61]. Although an unspecific Mac-1 blockade might, therefore, in theory, also predispose towards infection, we argue that the risk can be significantly reduced by specifically targeting only the activated receptor conformation (as with F7) leaving the functions of the non-activated Mac-1 receptor intact. In the various mouse models performed, mice that received F7 showed no signs of infection and did not develop weight loss compared to controls indicating that blocking-activated Mac-1 is well tolerated.

We demonstrated that F7 binds specifically to the human α_M_ I-domain of Mac-1 and to activated human monocytes and neutrophils and as such represents an interesting diagnostic tool. To assess the suitability of DARPin F7 for the measurement of monocyte activation in the clinic, we investigated patients in whom identification of inflammatory reaction could be helpful. Current markers of inflammation (CRP, white blood cell count etc.) are nonspecific and increases are often observed too late, after severe inflammation has already set in. As opposed to CRP, Mac-1 conformational change occurs within <1 s of monocyte activation [72] and could serve as a rapid marker of inflammation, enabling the identification of patients at risk. Elevated levels of inflammation, as determined by CRP, have been associated with poor outcomes after myocardial infarction [11], but a rise in CRP levels after myocardial infarction is usually delayed, peaking after 50 h [18].

We, however, have demonstrated increased monocyte activation as determined by increased binding of F7 to monocytes in STEMI patients within 36 h of PCI. Our results are in line with data from other groups which have detected increased monocyte activation after myocardial infarction by different means [66].

Furthermore, in addition to the ability to identify monocyte activation after myocardial infarction, F7 as a therapeutic agent may also be able to reduce inflammation contributing to cardiac ischaemia/reperfusion injury as shown in a mouse model of LAD ligation and reperfusion. The multifactorial harmful processes developing after reperfusion of the myocardium have been difficult to target therapeutically [33, 35, 37]. Our data are in line with previous studies which found anti-inflammatory strategies hold promise towards reducing ischaemia–reperfusion injury [51, 84]. Targeting activated Mac-1 on monocytes and neutrophils may, therefore, expand the anti-inflammatory therapeutic options for the prevention of cardiac ischaemia/reperfusion injury.

A patient cohort associated with the potential risk of severe inflammatory reactions are patients on ECMO, an extracorporeal circulatory support system that is increasingly used as a last resort for patients with respiratory and/or circulatory failure. Increased monocyte activation has been described in these patients [31]. Moreover, ECMO itself can trigger inflammatory reactions, which is independent of the underlying medical condition and is associated with worse outcome [53]. This ECMO-induced systemic inflammatory response syndrome is initiated by complementary activation upon blood contact with foreign surfaces, which later leads to leukocyte activation [53]. Increased Mac-1 expression on leukocytes has been reported in ECMO patients and could serve as a method for rapid assessment of immune activation in these patients [26]. We showed increased binding of F7 to activated monocytes in ECMO patients as compared to healthy controls.

F7 was also able to detect activated Mac-1 on monocytes in patients with pulmonary sepsis where early detection is crucial for survival. Previously, Eisenhardt et al. showed that the activation-specific anti-Mac-1 single-chain antibody MAN-1 could identify septic patients in the intensive care unit through its binding to activated monocytes [25]. In another cohort, MAN-1 was able to detect a reduction in monocyte activation after renal denervation in severely hypertensive patients [83]. CBRM1/5, on the other hand, only recognizes a subset of activated leukocytes [21], which does not support its application as a diagnostic tool.

A central disadvantage of MAN-1 and CBRM1/5 is, however, that they do not show binding to mouse Mac-1, which prevents testing of these antibodies as potential anti-inflammatory drugs in classical murine models of disease. F7, on the other hand, demonstrates binding to both mouse and human Mac-1, allowing broad and thorough testing for diagnostic and therapeutic use in mouse models. As in vivo testing in animal models is a prerequisite, especially for drug development, F7 is well placed for a drug-development program.

Moreover, F7 can be used to detect leukocyte activation in multiple inflammatory conditions. For instance, it was reported that Mac-1 expression correlates with disease activity in granulomatosis with polyangiitis [56]. Mac-1 expression was also increased on monocytes in patients with rheumatoid arthritis [45], in patients after percutaneous coronary intervention who developed restenosis [38] and in patients with sickle-cell disease [39].

DARPins offer several advantages over immunoglobulins, which were mentioned earlier. Potential disadvantages should also be considered, particularly the issue of immunogenicity. DARPins could potentially cause allergic reactions in humans as they present foreign antigens. Recent phase II clinical trials have, however, reported safe systemic application, e.g. the drug candidate MP0250, an anti-VEGF/HGF DARPin for the treatment of multiple myeloma [70]. Moreover, mice receiving DARPins in our preclinical models did not show any symptoms of allergic reactions or die prematurely. Additionally, like immunoglobulins, DARPins share the potential disadvantage of being protein therapeutics, which requires parenteral application. However, antibody or similar drugs are for some time now attracting the majority of pharmaceutical research investment and subcutaneous applications with weekly or even monthly injection intervals appear particularly attractive [48].

In conclusion, we were able to select a DARPin, which selectively binds and blocks the I-domain of Mac-1 and thereby specifically identifies and inhibits activated monocytes and neutrophils, both in mice and in humans. DARPin F7 shows highly promising therapeutic effects in mouse models of inflammation and can detect monocyte activation in patients.

## Supplementary Information

Below is the link to the electronic supplementary material.

Online Resource 1 (PDF 592 KB)

Online Resources 2–18 (PDF 1228 KB)

Online Resource 13 (MP4 10352 KB)

Online Resource 14 (MP4 10401 KB)

Online Resource 15 (MP4 10327 KB)

## Data Availability

All data are available from the authors upon reasonable request.

## Code availability

Only commercially available software was used in this research project and is given in the “Methods” section. Custom code was not used.
