# Supplementary material for: A DARPin targeting activated Mac-1 is a novel diagnostic tool and potential anti-inflammatory agent in myocarditis, sepsis and myocardial infarction
Source: Basic Res Cardiol. 2021 Mar 15;116(1):17. doi: 10.1007/s00395-021-00849-9 (PMC7960600; doi:10.1007/s00395-021-00849-9)
Supplement: Supplementary file 1 — Online Resource 1 (PDF 592 KB) [file 395_2021_849_MOESM1_ESM.pdf]

A DARPin targeting activated Mac-1 is a novel diagnostic tool and potential anti-inflammatory agent in myocarditis, sepsis and myocardial infarction

Patrick M Siegel, MD[1,2*]; István Bojti, MD[1*]; Nicole Bassler[2]; Jessica Holien, PhD[3]; Ulrike Flierl, MD[2]; Xiaowei Wang, PhD[2,4,5]; Philipp Waggershauser[1]; Xavier Tonnar, MD[1]; Christopher Vedecnik, MD[1]; Constanze Lamprecht, PhD[6]; Ivana Stankova, MD[1]; Tian Li, MD[1]; Thomas Helbing, MD[1]; Dennis Wolf, MD[1]; Nathaly Anto-Michel, PhD[1]; Lucia Sol Mitre, PhD[1]; Julia Ehrlich, MD[1]; Lukas Orlean[1]; Ileana Bender[1]; Anne Przewosnik[1]; Maximilian Mauler, PhD[1]; Laura Hollederer[1]; Martin Moser, MD[1]; Christoph Bode, MD[1]; Michael W Parker, PhD[3,4,7]; Karlheinz Peter, MD, PhD[2,4,5§], Philipp Diehl, MD, PhD[1,2,5§]

[1] Cardiology and Angiology I, Heart Center Freiburg University, Faculty of Medicine, University of Freiburg, Freiburg, Germany

[2] Atherothrombosis and Vascular Biology Laboratory, Baker Heart and Diabetes Institute, Melbourne, Australia

[3] ACRF Rational Drug Discovery Centre, St. Vincent's Institute of Medical Research, Melbourne, Australia

[4] Baker Department of Cardiometabolic Health, University of Melbourne, Melbourne, Australia

[5] Department of Medicine, Central Clinical School, Monash University, Melbourne, Australia

[6] BIOSS Centre for Biological Signalling Studies/Synthetic Biology of Signalling Processes, University of Freiburg, Freiburg, Germany

[7] Bio21 Molecular Science and Biotechnology Institute, University of Melbourne, Melbourne, Australia

*equally contributing first authors, [§]equally contributing senior authors

Corresponding author

Karlheinz Peter, MD, PhD

Baker Heart and Diabetes Institute

75 Commercial Road, Melbourne VIC 3004, Australia

Email: karlheinz.peter@baker.edu.au

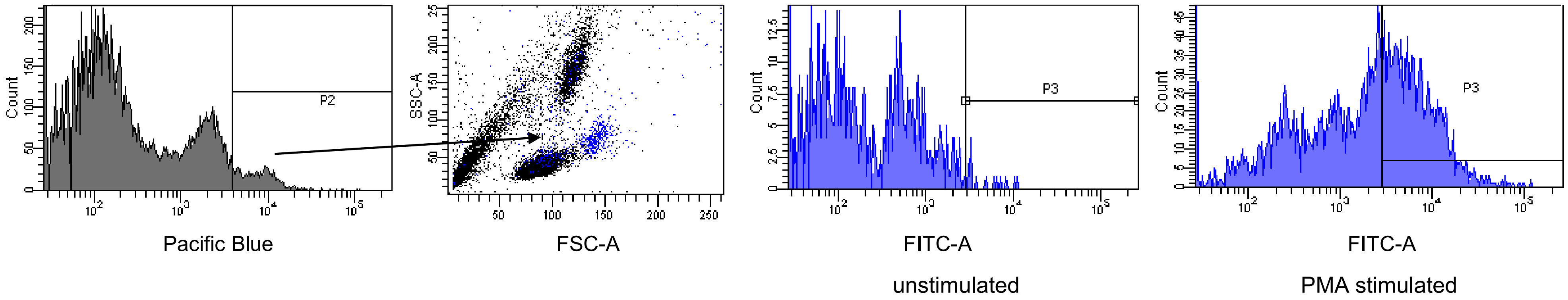

Gating of the top 1-3% of P2 = Ly-6C++ cells, distinct population

Backgating to FSC/SSC verified monocyte location

P3 = DARPin binding in percentage of Ly-6C++ monocytes. P3 was set as the top 1-3% of the unstimulated population stained only with the secondary Alex Fluor 488 antibody

Whole blood FACS assay - mouse monocytes (Fig 2c & 3c)

After stimulation with PMA or PBS (unstimulated samples) lysed mouse blood was stained with a Pacific Blue anti-Ly-6C antibody (Biolegend, USA), DARPins and a secondary Alexa Fluor 488 anti-His-tag antibody (Qiagen, Germany). In a histogram displaying Pacific Blue positive cells, the top 1-3% positive cells (always a distinct population) were gated. Backgating was performed to the FSC/SSC gate to confirm location of these cells in the expected monocyte gate (blue). Binding of these Ly-6C++ highly positive monocytes was assessed in percent in a FITC histogram. To define the positive population, a gate was set in the FITC histogram including the upper 1-3 % of the unstimulated monocyte population stained with the secondary anti-His antibody. The monocytes stained with DARPins and the secondary antibody shifting into this gate were then recorded in percentages. Analysis was performed using FACS DIVA V6.1 (Becton Dickinson, USA)

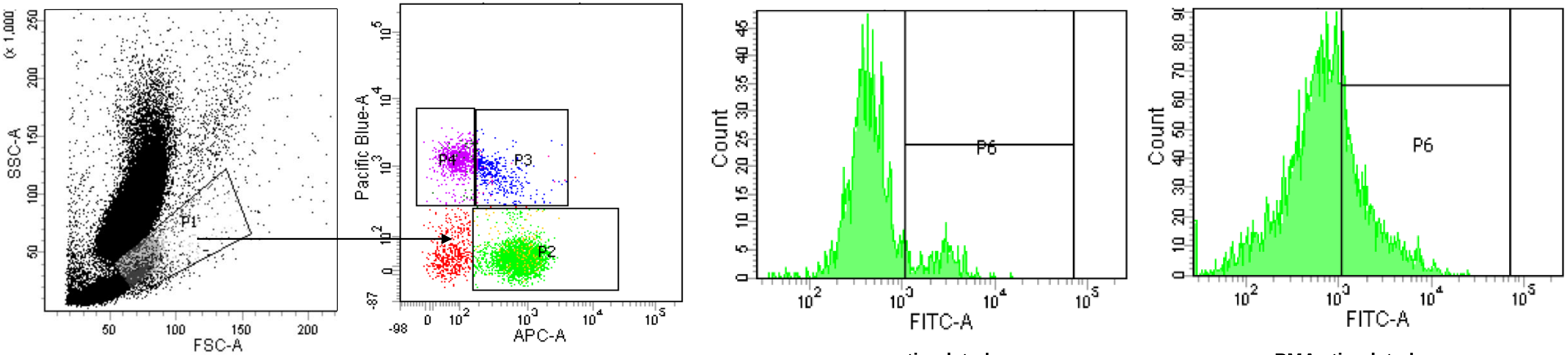

unstimulated **PMA stimulated**

Whole blood FACS assay – human monocytes (Fig 6 c-e, g)

After stimulation with PMA or PBS (unstimulated samples) lysed human blood was stained with Pacific Blue anti-CD16 and APC anti-CD14 antibodies (Both, Biolegend, USA), DARPins and a secondary Alexa Fluor 488 anti-His-tag antibody. Monocytes were pre-identified according to their location on the FSC/SSC plot (P1). According to their CD14 and CD16 expression, monocytes were then categorized as classical (P2), intermediate (P3) or non-classical (P4) monocytes. Classical monocytes were then depicted in a FITC histogram to record DARPin binding. To define the positive population, a gate (P6) was set in the FITC histogram including the upper 1-3 % of the unstimulated monocyte population stained with the secondary Alexa Fluor 488 anti-His-tag antibody (Qiagen, Germany). The monocytes stained with DARPins and the secondary antibody shifting into this gate were then recorded in percentages. Analysis was performed using FACS DIVA V6.1 (Becton Dickinson, USA). Presentation of histograms of Fig. 6d was prepared using FlowJo V10.6

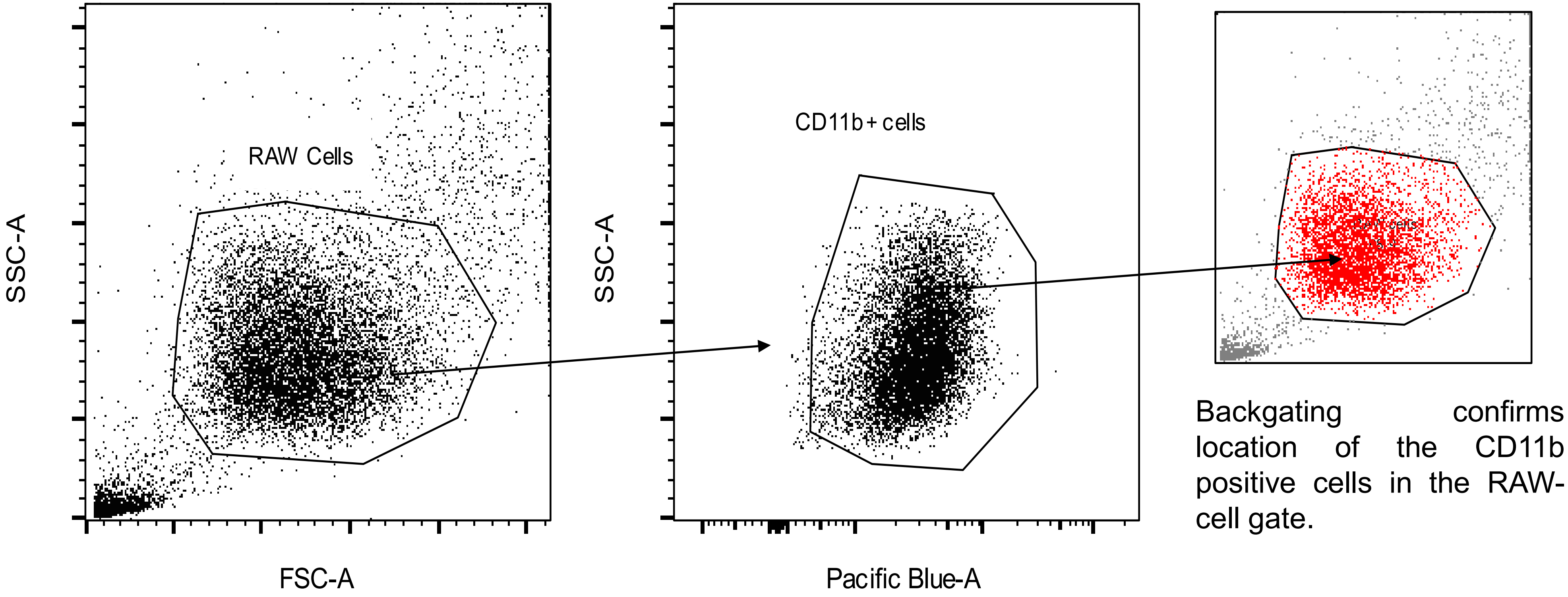

DARPin binding to RAW 264.7 cells and isolated mouse monocytes (Fig. 2b, Fig. 2d)

After isolation cells were stained with DARPins and the Alexa Fluor 488 anti-His-tag antibody. Gating was performed according to FSC/SSC plots. To confirm CD11b expression on RAW 264.7 cells one sample was always stained with a Pacific blue anti-CD11b (Biolegend, USA) antibody. DARPin binding was recorded in mean fluorescence intensity (MFI) FITC

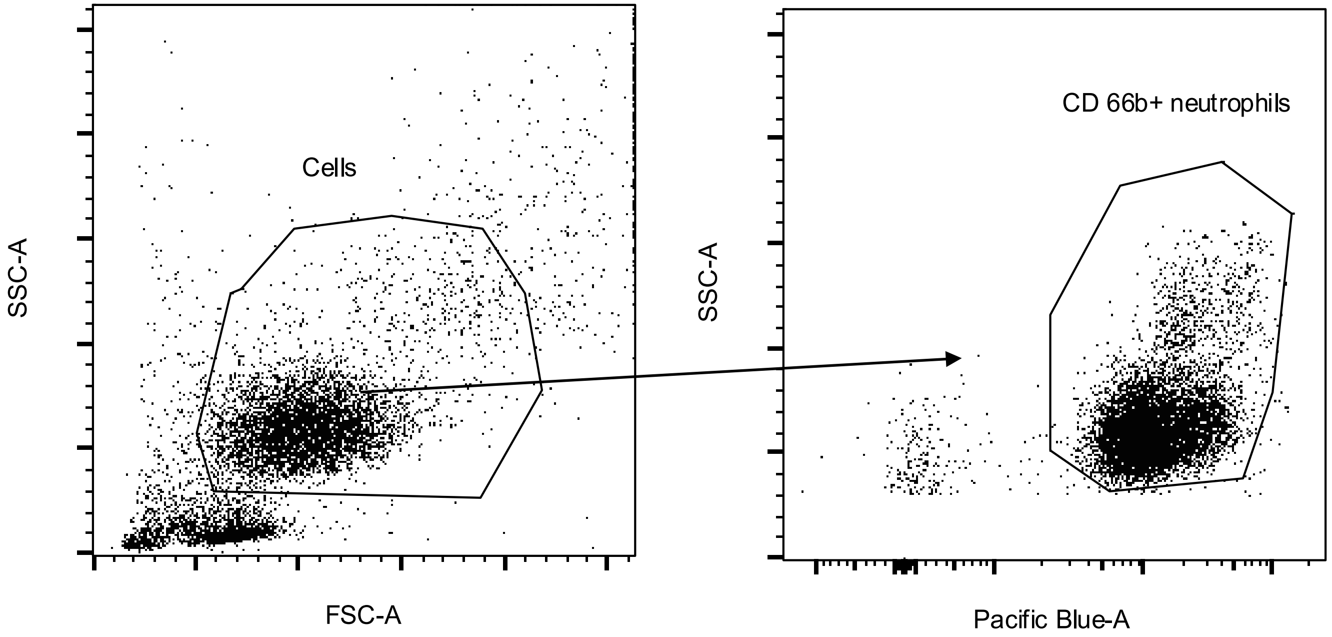

DARPin binding to human neutrophils (Fig. 6f)

After isolation by density gradient centrifugation, neutrophils were identified by Pacific-Blue anti-CD66b antibody (Biolegend, USA) staining in the Pacific Blue/SSC gate. DARPins and the Alexa Fluor anti-His-tag secondary antibody were added after 2 min of acquisition. Binding to DARPin F7 (detected via secondary Alexa Fluor 488 anti-His-Tag antibody (Qiagen, Germany) was quantified over time using the Flowjo Kinetics function

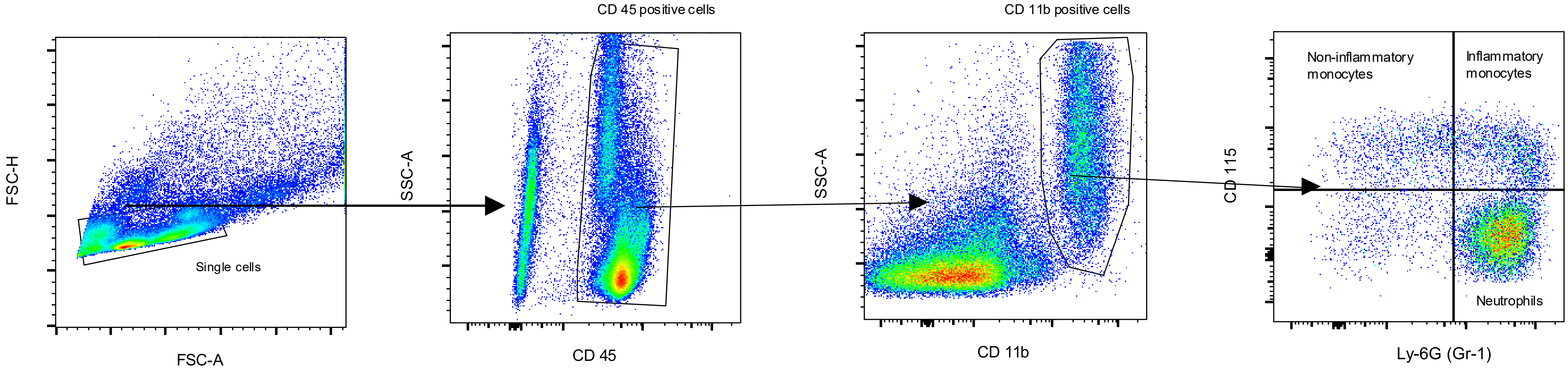

Gating strategy – CLP model (Fig. 4h-o)

After blood taking and harvest the peritoneal lavage, cells were stained with a mastermix containing a Pacific Blue anti-CD45 antibody, a FITC anti-Mac-1 antibody, a PE-Cy7 anti-Ly-6G (Gr-1) antibody, an APC anti-CD115 antibody and and a PE anti-F4/80 antibody. First, $CD45^+$ cells were identified. From there on the following gating strategy was applied. The gating strategy for blood monocytes is presented as an example above.

- $CD45^+$ $CD11b^+$ $CD115^+$ Monocytes
- $CD45^+$ $CD11b^+$ $CD115^+$ Ly-6G $(Gr\text{-}1)^+$ Inflammatory monocytes
- $CD45^+$ $CD11b^+$ $CD115^+$ Ly-6G $(Gr\text{-}1)^-$ Non-inflammatory monocytes
- $CD45^+$ $CD11b^+$ $CD115^-$ Ly-6G $(Gr\text{-}1)^+$ Neutrophils
- In the peritoneal lavage (not shown), macrophages were additionally identified as: $CD45^+$ $CD11b^+$ Ly-6G $(Gr\text{-}1)^-$ $F4/80^+$
- Total cell count was quantified using an automated cell counter and is given per µl. Absolute numbers of different cell populations were derived from their percentage of the total cell count. Analysis was performed using FlowJo V10.0 software

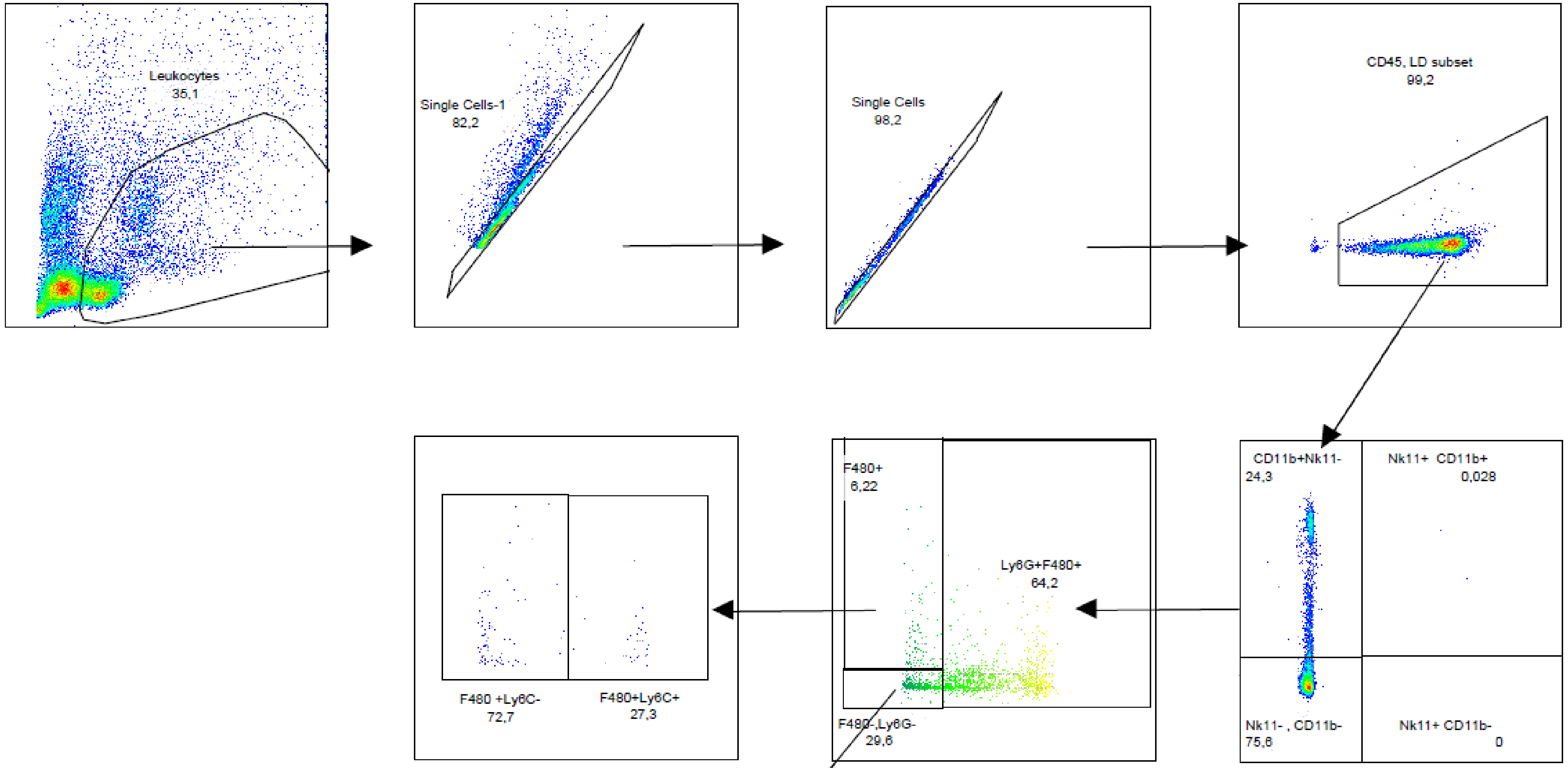

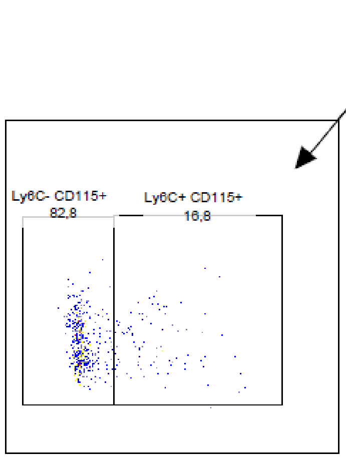

Gating strategy – EAM Model (Online Resource 9)

After harvesting the heart of EAM mice and lysis or blood taking cells were stained with a mastermix containing:

Anti-CD45 PerCP/Cy5.5

Anti-CD11b PE-Cy7

Anti-F4/80 PE

Anti-Ly-6G AF 700

Anti-Nk1.1 BV650

Anti-Ly-6C BV 785

Anti CD115 APC

First, $CD45^+$ cells were identified. From there on the gating strategy (left) was applied.

Total cell count was quantified using an automated cell counter and is given per µl of blood or peritoneal lavage. Absolute numbers of different cell populations were derived from their percentage of the total cell count. Analysis was performed using FlowJo V10.0 software.

The most important cells for our study that were identified were:

• $CD45^+$ $CD11b^+$ $Nk1.1^-$ $Ly\text{-}6G^-$ $F4/80^-$ $CD115^+$	Monocytes
• $CD45^+$ $CD11b^+$ $Nk1.1^-$ $Ly\text{-}6G^-$ $F4/80^-$ $CD115^+$ $Ly\text{-}6C^+$	Inflammatory monocytes
• $CD45^+$ $CD11b^+$ $Nk1.1^-$ $Ly\text{-}6G^-$ $F4/80^-$ $CD115^+$ $Ly\text{-}6C^-$	Non-inflammatory monocytes
• $CD45^+$ $CD11b^+$ $Nk1.1^-$ $Ly\text{-}6G^+$ $F4/80^+$ $CD115^-$	Neutrophils
• $CD45^+$ $CD11b^+$ $Nk1.1^-$ $Ly\text{-}6G^-$ $F4/80^+$ $Ly\text{-}6C^{+/-}$	Macrophages
