# Supplementary material for: A DARPin targeting activated Mac-1 is a novel diagnostic tool and potential anti-inflammatory agent in myocarditis, sepsis and myocardial infarction
Source: Basic Res Cardiol. 2021 Mar 15;116(1):17. doi: 10.1007/s00395-021-00849-9 (PMC7960600; doi:10.1007/s00395-021-00849-9)
Supplement: Supplementary file 2 — Online Resources 2–18 (PDF 1228 KB) [file 395_2021_849_MOESM2_ESM.pdf]

A DARPin targeting activated Mac-1 is a novel diagnostic tool and potential anti-inflammatory agent in myocarditis, sepsis and myocardial infarction

Patrick M Siegel, MD[1,2*]; István Bojti, MD[1*]; Nicole Bassler[2]; Jessica Holien, PhD[3]; Ulrike Flierl, MD[2]; Xiaowei Wang, PhD[2,4,5]; Philipp Waggershauser[1]; Xavier Tonnar, MD[1]; Christopher Vedecnik, MD[1]; Constanze Lamprecht, PhD[6]; Ivana Stankova[1]; Tian Li[1]; Thomas Helbing, MD[1]; Dennis Wolf, MD[1]; Nathaly Anto-Michel, PhD[1]; Lucia Sol Mitre, MD[1]; Julia Ehrlich[1]; Lukas Orlean[1]; Ileana Bender[1]; Anne Przewosnik[1]; Maximilian Mauler, PhD[1]; Laura Hollederer[1]; Martin Moser, MD[1]; Christoph Bode, MD[1]; Michael W Parker, PhD[3,4,7]; Karlheinz Peter, MD, PhD[2,4,5§], Philipp Diehl, MD, PhD[1,2,5§]

[1] Cardiology and Angiology I, Heart Center Freiburg University, Faculty of Medicine, University of Freiburg, Freiburg, Germany

[2] Atherothrombosis and Vascular Biology Laboratory, Baker Heart and Diabetes Institute, Melbourne, Australia

[3] ACRF Rational Drug Discovery Centre, St. Vincent's Institute of Medical Research, Melbourne, Australia

[4] Baker Department of Cardiometabolic Health, University of Melbourne, Melbourne, Australia

[5] Department of Medicine, Central Clinical School, Monash University, Melbourne, Australia

[6] BIOSS Centre for Biological Signalling Studies/Synthetic Biology of Signalling Processes, University of Freiburg, Freiburg, Germany

[7] Bio21 Molecular Science and Biotechnology Institute, University of Melbourne, Melbourne, Australia

*equally contributing first authors, [§]equally contributing senior authors

Corresponding author

Karlheinz Peter, MD, PhD

Baker Heart and Diabetes Institute

75 Commercial Road, Melbourne VIC 3004, Australia

Email: karlheinz.peter@baker.edu.au

Mouse I-domain DNA sequence

```
CC
ATG GTC CTG AGG CCG CCC CAG CAG TTC CCA GAG GCT CTC AGA GAA TGT
CCT CAG CAG GAG AGT GAC ATT GTC TTC TTG ATT GAT GGC TCC GGT AGC
ATC AAC ARC ATT GAC TTT CAG AAG ATG AAG GAG TTT GTC TCA ACT GTG
ATG GAG CAG TTC AAA AAG TCT AAA ACC TTG TTC TCT TTG ATG CAG TAC
TCG GAC GAG TTC CGG ATT CAC TTC ACC TTC AAT GAC TTC AAG AGA AAC
CCT AGC CCA AGA TCA CAT GTG AGC CCC ATA AAG CAG CTG AAT GGG AGG
ACA AAA ACT GCC TCA GGG ATC CGG AAA GTA GTG AGA GAA CTG TTT CAC
AAA ACC AAT GGG GCC CGG GAG AAT GCT GCG AAG ATC CTA GTT GTC ATC
ACA GAT GGA GAA AAA TTC GGT GAT CCC TTG GAT TAT AAG GAT GTC ATC
CCC GAG GCA GAC AGA GCA GGG GTC ATT CGC TAC GTA ATT GGG GTG GGA
AAT GCC TTC AAC AAA CCA CAG TCC CGC AGA GAG CTC GAC ACC ATC GCA
TCT AAG CCA GCT GGT GAA CAC GTG TTC CAA GTG GAC AAC TTT GAA GCC
CTG AAT ACC ATT CAG AAC CAG CTT CAG GAA AAG ATC TTT GCA ATT GAG
GGC ACG CAG ACA GGA AGT ACC AGC TCC TTT GAG CAT GAG ATG TCT CAA
GAA GGC TTC AGT GCT TCC CGA GGA GGG CCC GAA CAA AAA CTC ATC TCA
GAA GAG GAT CTG AAT AGC GCC GTC GAC CTG CCT GAG ACT GGC GGG GAG
GCG GCC GC
```

Mouse I-domain amino acid sequence

```
M V L R P P Q Q F P E A L R E C P Q Q E S D I V F L I D G S G S I
N N I D F Q K M K E F V S T V M E Q F K K S K T L F S L M Q Y S D
E F R I H F T F N D F K R N P S P R S H V S P I K Q L N G R T K T
A S G I R K V V R E L F H K T N G A R E N A A K I L V V I T D G E
K F G D P L D Y K D V I P E A D R A G V I R Y V I G V G N A F N K
P Q S R R E L D T I A S K P A G E H V F Q V D N F E A L N T I Q N
Q L Q E K I F A I E G T Q T G S T S S F E H E M S Q E G F S A S R
G G P E Q K L I S E E D L N S A V D L P E T G G E A A A
```

Restriction sites:

NcoI

NotI

Tags included in sequence

LPETG tag

c-myc tag

Mutation sites:

1st Model: Mouse I-domain mutation sites: N146A, E179A, N206A

2nd Model: Mouse I-domain mutation sites: R208A, F246D

"Activated mouse I-domain, or a-mId": F302L

Online Resource 2 DNA and amino acid sequences of the mouse I-domain of Mac- 1. Sequences are presented within the restriction sites before cloning into the vector. Mutation sites, restriction sites for restriction enzymes and protein tags included are presented by colour coding

DARPin F7 DNA sequence

GGA TCC GAC CTG GGT AAG AAA CTG CTG GAA GCT GCT CGT GCT GGT CAG
GAC GAC GAA GTT CGT ATC CTG ATG GCT AAC GGT GCT GAC GTT AAC GCT
ATT GAC TTT TTT GGT TCT ACT CCG CTG CAC CTG GCT GCT GAG CTT GGT
CAC CTG GAA ATC GTT GAA GTT CTG CTG AAG AAC GGT GCT GAC GTT AAC
GCT GAT GAC ATT CTT GGT GAT ACT CCG CTG CAC CTG GCT GCT GAT TTT
GGT CAC CTG GAA ATC GTT GAA GTT CTG CTG AAG AAC GGT GCT GAC GTT
AAC GCT TCT GAC GCT TTT GGT CTT ACT CCG CTG CAC CTG GCT GCT AAT
GCT GGT CAC CTG GAA ATC GTT GAA GTT CTG CTG AAG CAC GGT GCT GAC
GTT AAC GCT CAG GAC AAA TTC GGT AAG ACC GCT TTC GAC ATC TCC ATC
GAC AAC GGT AAC GAG GAC CTG GCT GAA ATT CTG CAA AAG CTT

DARPin F7 amino acid sequence

Capping structure
G S D L G K K L L E A A R A G Q D D E V R I L M A N G A D V N A
1st repeat
I D F F G S T P L H L A A E L G H L E I V E V L L K N G A D V N A
2nd repeat
D D I L G D T P L H L A A D F G H L E I V E V L L K N G A D V N A
3rd repeat
S D A F G L T P L H L A A N A G H L E I V E V L L K H G A D V N A
Capping structure
Q D K F G K T A F D I S I D N G N E D L A E I L Q K L N

Restriction sites
Bam HI
HindIII

Mutation sites
E46A
D71A
D79A

Online Resource 3 DNA and amino acid sequences of DARPin F7. Sequences are presented within the restriction sites before cloning into the vector. Mutation sites, restriction sites for restriction enzymes and protein tags included are presented by colour coding. DARPins are composed of a C- and N-terminal capping structure and different numbers of variable repeats. DARPin F7 has 3 variable repeats

Clinical characteristics of ECMO patients

Total No. of patients	16
Male : Female	7 : 9
Age in years (Mean ± SD)	56.7 ± 16.7
Survival to discharge (% of total)	7 (43.75)
VA-ECMO for cardiogenic shock<br>due to<br>- Ischemic<br>- Tachymyopathy<br>- Heart Failure	8<br><br>4<br>2<br>2
VV-ECMO for ARDS<br>due to<br>- Influenza pneumonia<br>- PJP<br>- Bacterial pneumonia	8<br><br>4<br>3<br>1
Acute renal failure during ECMO	8
Acute liver failure during ECMO	4
Resuscitation before and during time on ECMO	6
Hemoglobin (g/dL) (Mean ± SD)	8.6 ± 1.6
Leukocyte count per µL (Mean ± SD)	16601 ± 9447
Platelet count per µL (Mean ± SD)	118133 ± 85933
CRP (mg/dL) (Mean ± SD)	151.7 ± 102.6
Creatinine (mg/dL) (Mean ± SD)	1.5 ± 0.75

Online Resource 4 Clinical characteristics of ECMO patients. This tables shows an overview of relevant clinical characteristics of the ECMO patients assessed in this study. Laboratory parameters on the day of blood withdrawal for assessment of DARPin binding are presented. ARDS – acute respiratory distress syndrome, CRP – C-reactive protein, ECMO – extra-corporeal membrane oxygenation, PJP - Pneumocystis jirovecii pneumonia, SD – standard deviation, VA- veno-arterial, VV – veno-venous

Clinical characteristics of STEMI patients

Total No. of patients	17
Male: Female	15 : 2
Age in years (Mean ± SD)	61.7 ± 9.2
Cardiovascular risk factors	No. of patients
Hypercholesterolemia	3
Smoking	9
Hypertension	6
Obesity	1
Diabetes mellitus	1
Culprit lesion (Artery: # of patients)	LAD: 8<br>LCX:3<br>RCA: 6
No. of Stents (Mean ± SD)	1.643 ± 0.75
BMI (Mean ± SD)	25.33 ± 3.30
Hours to Blood taking (Mean ± SD)	21.46 ± 6.42
CK Max (U/L) (Mean ± SD)	1880 ± 1588
CRP (mg/L) (Mean ± SD)	16.92 ± 36.40
Leukocyte count per µL (Mean ± SD)	10740 ± 2808
Platelet count per µL (Mean ± SD)	211643 ± 58859
Creatinine (mg/dL) (Mean ± SD)	0.86 ± 0.16
LVEF (%) before discharge (Mean ± SD)	44.29 ± 11.58

Online Resource 5 Clinical characteristics of STEMI patients. This table shows an overview of relevant clinical characteristics of the STEMI patients assessed in this study Laboratory parameters on the day of blood withdrawal for assessment of DARPin binding are presented. BMI – Body mass index, CK – creatine kinase, CRP – C-reactive Protein, No. – Number, LVEF – left ventricular ejection fraction, LAD – left anterior descending artery, LCX – left circumflex artery, RCA – right coronary artery, SD – standard deviation, STEMI – ST-elevation myocardial infarction

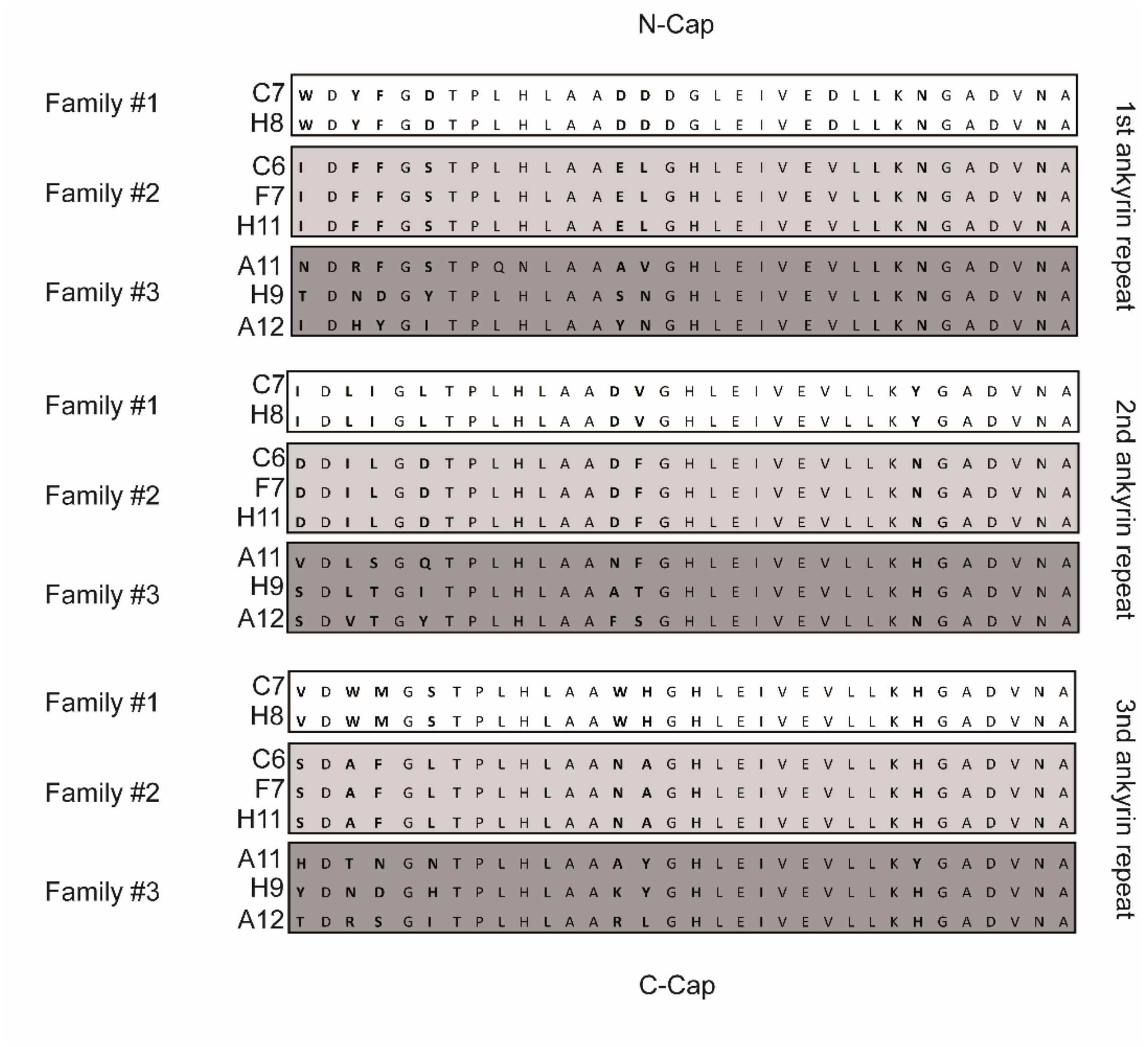

Online Resource 6. Overview of different DARPin families selected towards the α_M I-domain of mouse Mac-1. Selected anti-mouse α_M I-domain DARPin clone families. DARPin clones binding to mouse α_M I-domain were selected. Sequencing revealed that 3 different DARPin families were selected indicating specific panning on mouse α_M I-domain

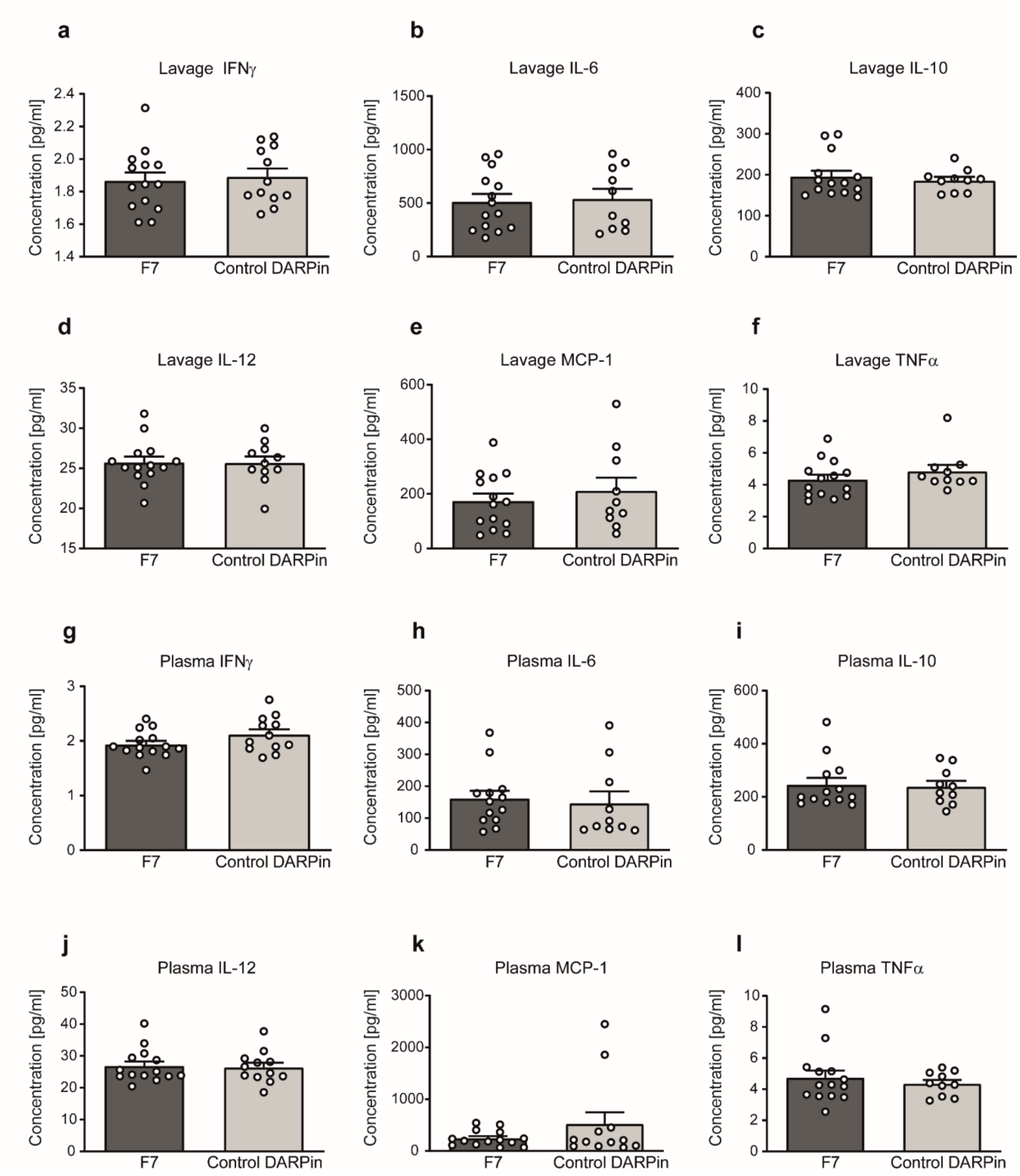

Online Resource 7 Cytokine levels in the peritoneal lavage (**a-f**) and plasma (**g-l**) of mice after cecal ligation and puncture. Blood and lavage were harvested 20 h after cecal ligation and puncture. Mice were treated with either DARPin F7 or the control DARPin intravenously 1 h before surgery. Cytokine plasma levels were determined by a cytometric bead array (CBA, BD Biosciences), according to the manufacturer's protocol. n=9-10 mice per treatment group. Data is presented as mean±SEM

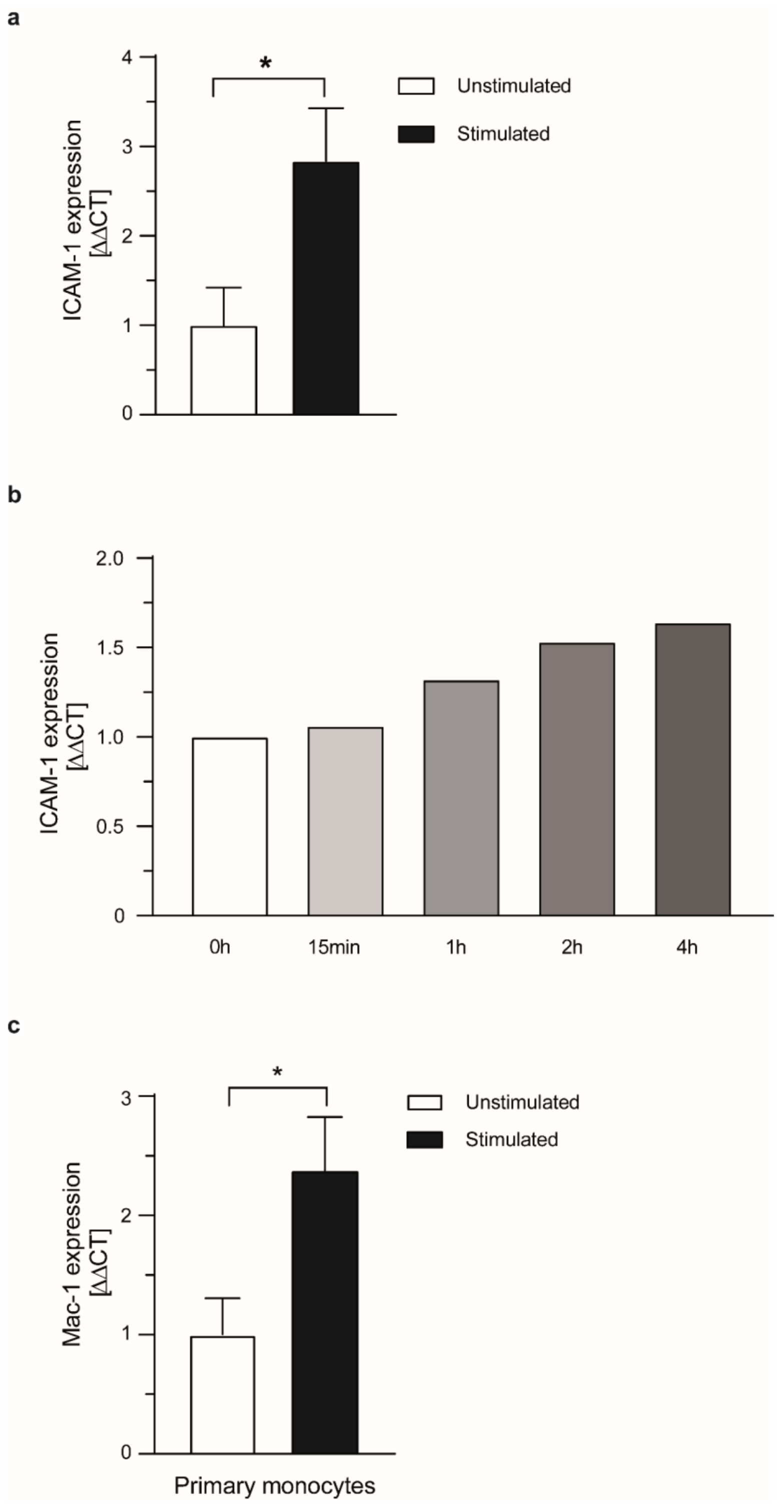

Online Resource 8. Expression of ICAM-1 by cardiomyocytes and Mac-1 by mouse monocytes. (**a**) Cells were isolated and stimulated as described in the methods section. Cardiomyocytes show increased expression of ICAM-1 when stimulated with TNFα (as determined by qPCR using the $\Delta\Delta C_t$ -method for analysis (n=5, $p<0.05$). (**b**) ICAM-1 expression in cardiomyocytes tends to increase over time in a proof-of-principle experiment. (**c**) Mouse monocytes show increased expression of Mac-1 after stimulation by 24h starvation compared to unstimulated mouse monocytes. Expression was determined by qPCR using the $\Delta\Delta C_t$ -method for analysis (n=5, *$p<0.05$). These results indicate that cardiomyocytes and monocytes express the adhesion molecules necessary for direct interaction

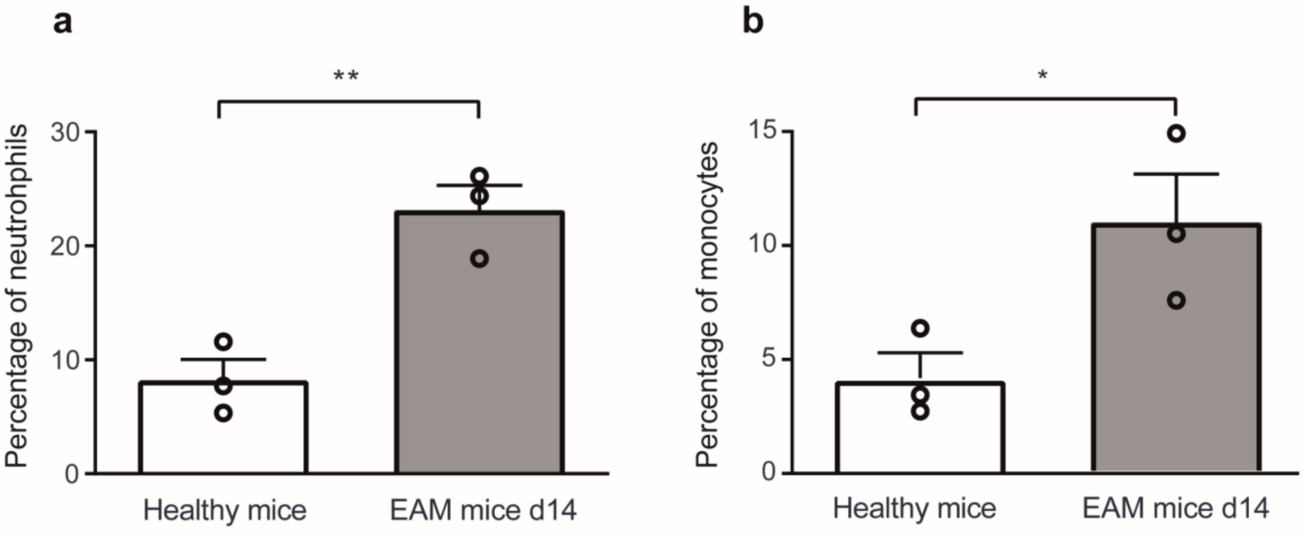

Online Resource 9 Neutrophils (**a**) and monocytes (**b**) in the myocardium of mice with experimental autoimmune myocarditis (EAM) compared to healthy control mice. Myocarditis was induced as described in the methods section. On day 14 (d14) EAM mice were euthanized and the heart was lysed and harvested and flow cytometry was performed. Monocytes were identified by $CD45^{+}$ $CD11b^{+}$ $Nk1.1^{-}$ $Ly\text{-}6G^{-}$ $F4/80^{-}$$CD115^{+}$ $Ly\text{-}6C^{+/-}$ expression, neutrophils were identified by $CD45^{+}$ $CD11b^{+}$ $Nk1.1^{-}$ $Ly\text{-}6G^{+}$ $F4/80^{+}$ $CD115^{-}$. Healthy mice were of the same age and weight, but myocarditis was not induced. They were euthanized on the same day as EAM mice. Data indicates that induction of myocarditis led to increased influx of myeloid cells. n=3 mice per group. *p<0.05, **p<0.01, p-values were calculated using an unpaired two-tailed Student's t-test. Data is presented as mean±SEM

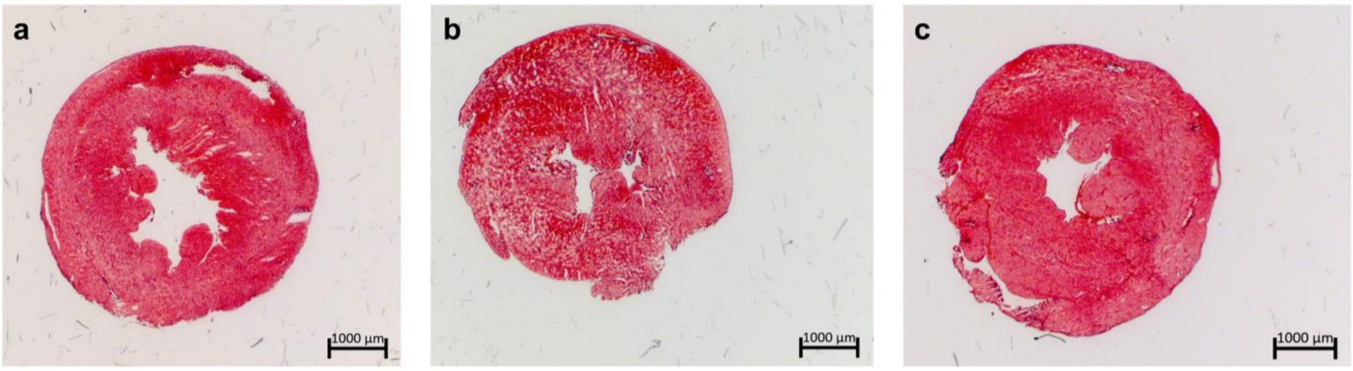

Online Resource 10 Representative histological sections of the heart of mice with EAM on day 50 stained with Masson's trichrome. Mice were treated either with F7 (**a**), the control DARPin (**b**) or PBS (**c**). Mice were euthanized on day 50 and after harvest of the heart histological sections were stained with Masson's trichrome according to standard protocols. Histological analysis showed no relevant fibrosis. The scale bar indicates 1000 µm

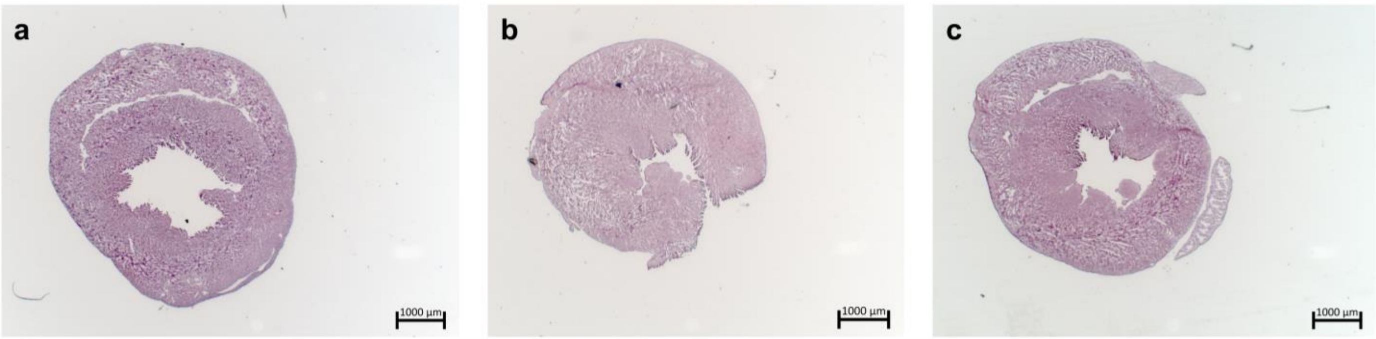

Online Resource 11 Representative histological sections of the heart of mice with EAM on day 50 stained with hematoxylin-eosin. Mice were treated either with F7 (**a**), the control DARPin (**b**) or PBS (**c**). Mice were euthanized on day 50 and after harvest of the heart histological sections were stained with hematoxylin-eosin according to standard protocols. HE staining showed no inflammatory infiltrates. The scale bar indicates 1000 µm

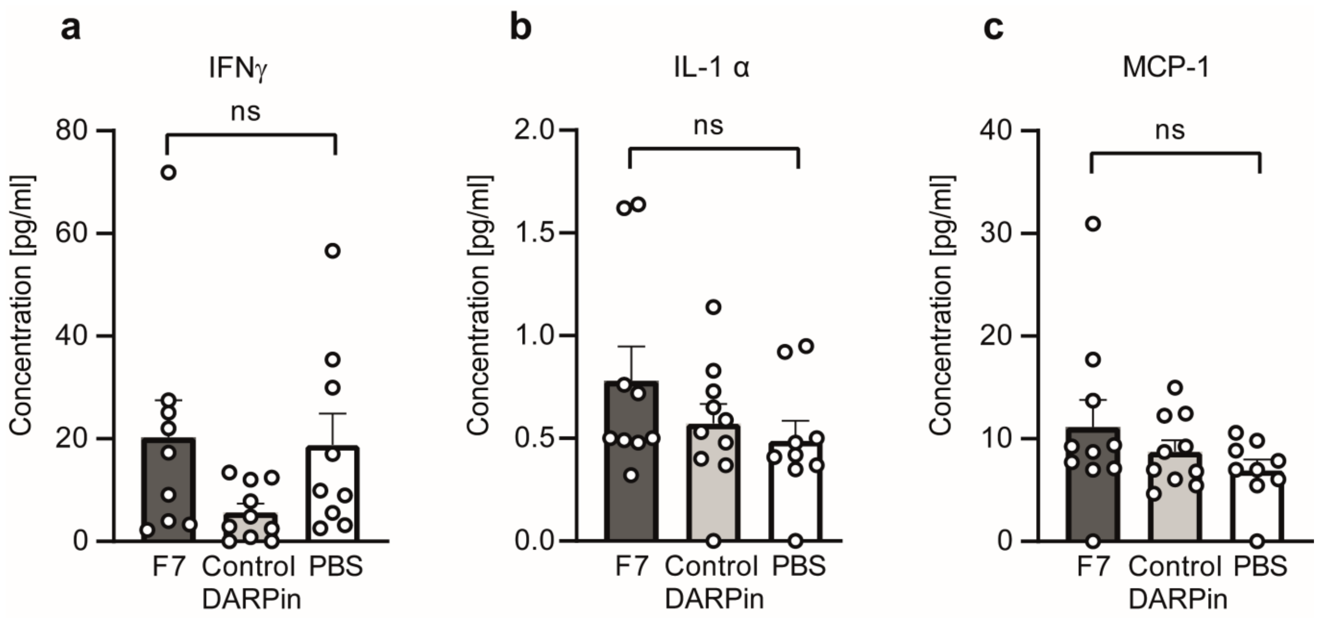

Online Resource 12 Cytokine levels in plasma of EAM mice. Myocarditis was induced and mice were treated with DARPin F7, the control DARPin or PBS as described in the methods section. Mice were euthanized on day 14 and blood was taken by cardiac puncture. Cytokine levels were determined using a "mix and match Legend plex Kit" (Biolegend, USA). It is a beads-based assay using fluorescence-encoded beads which are quantified flow-cytometrically. It was carried out according to the manufacturer's instructions. Levels of IFN-γ (**a**), IL-1α (**b**) and MCP-1 (**c**) were similar in all groups. n=10 per treatment group. p-values were calculated by one-way ANOVA. ns= not significant

Figure Captions for Online Resources 13-15

Online Resource 13 Representative echocardiographic loop of a mouse treated with DARPin F7. The video shows a representative echocardiographic loop of a C57BL/6 mouse which received DARPin for the treatment of myocarditis. Induction of myocarditis, treatments and image acquisition were performed as described in the methods section. Left ventricular ejection fraction was recorded on day 14 after myocarditis induction. LV-Function was nearly unaffected when mice were treated with F7

Online Resource 14 Representative echocardiographic loop of a mouse treated with the Control DARPin. The video shows a representative echocardiographic loop of a C57BL/6 mouse which received the Control DARPin for the treatment of myocarditis. Induction of myocarditis, treatments and image acquisition were performed as described in the methods section. Left ventricular ejection fraction was recorded on day 14 after myocarditis induction. LV-Function was significantly impaired in mice receiving the Control DARPin

Online Resource 15 Representative echocardiographic loop of a mouse treated with PBS. The video shows a representative echocardiographic loop of a C57BL/6 mouse which received PBS for the treatment of myocarditis. Induction of myocarditis, treatments and image acquisition were performed as described in the methods section. Left ventricular ejection fraction was recorded on day 14 after myocarditis induction. LV-Function was significantly impaired in mice receiving only PBS

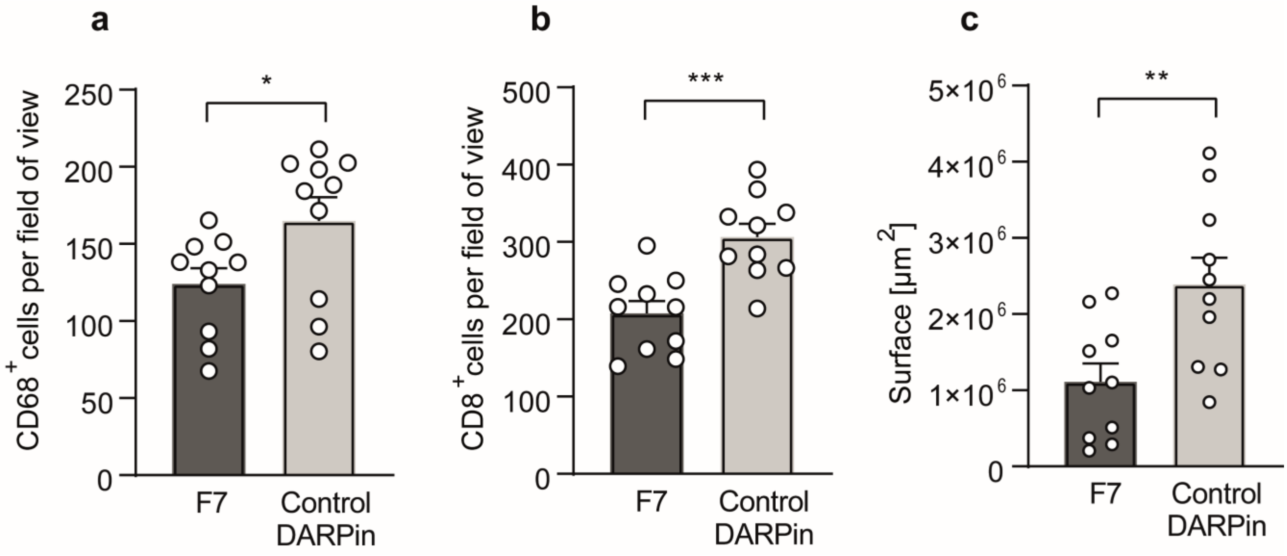

Online Resource 16 DARPin F7 reduces inflammation in a mouse model of 30 min coronary artery ligation and consequent reperfusion. Number of CD68+ cells (**a**) or CD8+ cells (**b**) per field at 20x magnification view as determined by immunohistochemistry and microscopic analysis. (**c**) Surface area of myocardial infarction as determined by HE-staining. Male C57BL/6 mice (Charles River, USA, 25-30g) received 200 µg of F7 or the control DARPin every 12 h from 12 h before surgery until euthanasia after 96 h. Mice were anesthetized with Ketamine/Xylazine by subcutaneous injection and surgery was described previously [9]. In brief, after placement on heating pad (37°C) and intubation mice were ventilated with and end-inspiratory pressure of 10 cm H2O at a respiratory rate of 110 breaths/min. Anesthesia was maintained with 1.2 % isoflurane. After left lateral thoracotomy, the left anterior descending artery (LAD) was identified and ligated with a loose 8-0 nylon suture for 30 min. Ischemia was verified visually by the change in color of the heart posterior to the ligation (red to gray). After 30 min the suture was removed, the pneumothorax evacuated and skin and thorax closed by suture. After surgery, mice received anesthesia with Buprenorphine subcutaneously every 6 h. After euthanasia at 96 h histology and microscopic analysis was performed as described for the EAM model with the exception of anti-CD8 immunohistochemistry. For this purpose, slides were stained with 50 µl of a rabbit anti-mouse anti-CD8 antibody (50 µl of 1:20 in PBS, 1 h, RT), followed by detection with a secondary goat anti-rabbit antibody (50 µl, 1:200 in PBS, 1h, RT). We found that treatment with F7 reduced numbers of $CD68^+$ macrophages and $CD8^+$ lymphocytes in the myocardium. Moreover, the area of myocardial infarction as determined by HE staining was smaller in mice receiving F7 compared to the control DARPin. Data is presented as mean±SEM; n=5 mice per treatment group. ns – not significant, *p<0.05, **p<0.01, ***p<0.001. p-values were calculated by a two-tailed unpaired Student’s t-test

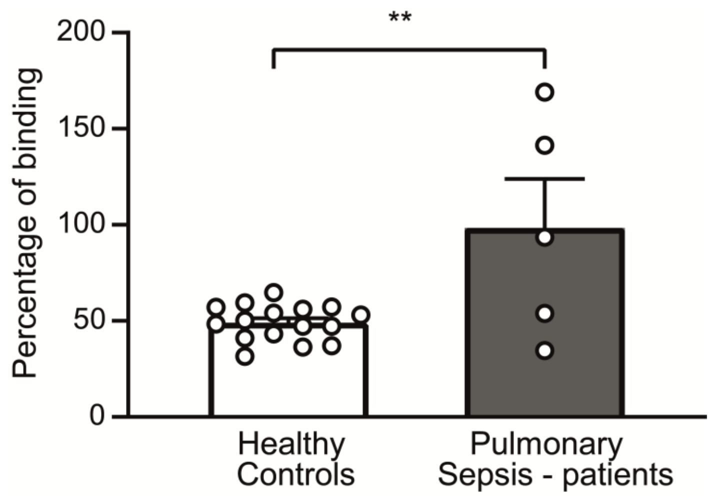

Online Resource 17 Binding of F7 to classical monocytes in patients with pulmonary sepsis and ARDS receiving ECMO (n=5) compared to the healthy group (n=16). Patients were hospitalized on our intensive care ward due to ARDS requiring ECMO-therapy caused by severe pulmonary infection requiring the use of antibiotics. All patients fulfilled the current “Sepsis-3” criteria for sepsis and septic shock. Citrated blood was taken within 24 h after ECMO initiation. The healthy controls are volunteers without severe pre-conditions that hadn’t taken any medication 2 weeks prior to blood taking (also shown in original Fig. 6 of the manuscript). Percentage of binding of DARPin F7 to $CD14^+$ monocytes was assessed by flow cytometry following the same methodology as with STEMI and ECMO patients provided in the methods section of our manuscript. We found a significantly higher percentage of binding in patients with pulmonary sepsis on ECMO. Data is presented as mean±SEM; **$p<0.01$, the p-value was calculated by a two-tailed unpaired Student’s t-test

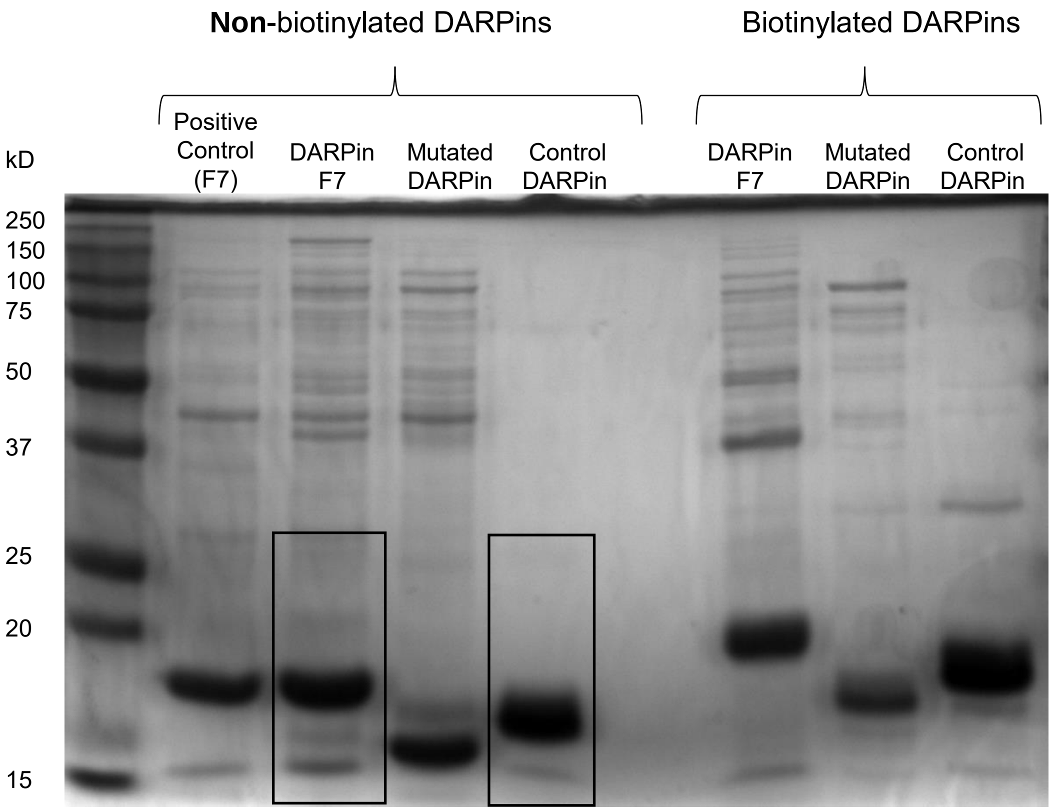

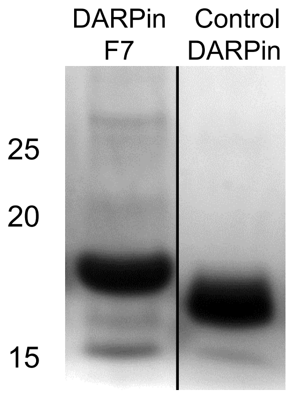

Online Resource 18. Full unedited gel of Fig. 1e. Please see the caption for Fig. 6 for further information
